# LRM3 positively regulates stem lodging resistance by degradating MYB6 transcriptional repressor in soybean

**DOI:** 10.1111/pbi.70124

**Published:** 2025-05-07

**Authors:** Yongheng Ye, Zhiyuan Cheng, Xinjing Yang, Suxin Yang, Kuanqiang Tang, Hui Yu, Jinshan Gao, Yaohua Zhang, Jiantian Leng, Wei Zhang, Ye Zhang, Moran Bu, Zhengwei Liang, Zhicheng Dong, Zhonghui Zhang, Xianzhong Feng

**Affiliations:** ^1^ Key Laboratory of Soybean Molecular Design Breeding, State Key Laboratory of Black Soils Conservation and Utilization Northeast Institute of Geography and Agroecology, Chinese Academy of Sciences Changchun China; ^2^ University of Chinese Academy of Sciences Beijing China; ^3^ Guangdong Provincial Key Laboratory of Plant Adaptation and Molecular Design, Guangzhou Key Laboratory of Crop Gene Editing, Innovative Center of Molecular Genetics and Evolution, School of Life Sciences Guangzhou University Guangzhou China; ^4^ Guangdong Provincial Key Laboratory of Biotechnology for Plant Development, School of Life Sciences South China Normal University Guangzhou China

**Keywords:** soybean, lodging resistance, LRM3, MYB6, PALs, lignin biosynthesis

## Abstract

Stem lodging resistance plays a critical role in maintaining soybean yield stability, yet the molecular mechanisms governing stem development and lodging tolerance remain poorly understood. Here, we report the characterization of *lodging‐related mutant 3* (*lrm3*), a weak‐stemmed soybean line exhibiting increased lodging susceptibility. Molecular cloning revealed that *LRM3* encodes a U‐box E3 ubiquitin ligase that physically interacts with the transcription factor MYB6, targeting it for 26S proteasome‐mediated degradation. Transcriptomic and chromatin immunoprecipitation analyses demonstrated that MYB6 binds directly to the promoter regions of *PHENYLALANINE AMMONIA‐LYASE* (*PAL*) genes, repressing their transcriptional activity and consequently reducing lignin biosynthesis and secondary cell wall deposition in stems. Population genetic analysis identified three major *LRM3* haplotypes, with Haplotype 1 preferentially retained in landraces and modern cultivars, suggesting artificial selection during domestication. Collectively, our findings elucidate a previously uncharacterized regulatory mechanism integrating ubiquitin‐mediated proteolysis and phenylpropanoid metabolism to enhance stem mechanical strength. This study provides novel genetic insights and molecular tools for improving lodging resistance in soybean breeding programs.

## Introduction

Lodging of soybean (*Glycine max* L.) plants is a limiting factor in cultivation and adversely affects yield. The legume soybean is a significant cash and oil crop, contributing to 59% of the world's oil production and providing 70% of the global plant protein for both humans and animals. In soybean, lodging can reduce yield at different growth stages by up to 12%–30% (Fehr *et al*., 1971; Sarkar *et al*., 2023). In rice and wheat, ‘Green Revolution’ genes *semi‐dwarf1 (sd1)* and *Reduced height (Rht)* reduced lodging tolerance and plant height through gibberellin metabolism to increase grain yield (Peng *et al*., 1999; Sasaki *et al*., 2002; Spielmeyer *et al*., 2002). However, soybean is a pod crop, and simply reducing plant height reduces the number of nodes, which results in fewer pods and reduced yield (Pedersen and Lauer, 2004). Therefore, obtaining lodging‐resistant plants is a potential strategy to increase soybean yield. This may be achieved by enhancing stem strength, thus allowing plants to adapt to high‐density planting (Liu *et al*., 2020). Lodging manifests by bending or buckling of stems during plant growth, which can be attributed to internal or external factors that impair proper stem growth. Lodging leads to reduced photosynthetic efficiency as a result of leaf contact with the ground and uneven distribution of light, thereby hindering the absorption and conversion of solar energy necessary for photosynthesis and growth. At the population level, spatial competition and resource‐use efficiency are also altered by occupying more soil space and consuming water and nutrients than are required for proper growth and development (Hussain *et al*., 2020; Yang *et al*., 2020). This ultimately results in a yield penalty and potential disparities in production across regions or fields.

Lignin, an organic compound present in the cell walls of plants, provides structural integrity and resistance to external stressors (Cesarino, 2019). By enhancing cell‐wall hardness and stability, lignin enables stems to withstand external mechanical pressures like wind, ensuring an upright growth habit typical of cultivated soybean varieties with high yields (Barros *et al*., 2019). This is particularly significant for crops at maturity and harvest when seeds are heavier and require robust stem support (Wang *et al*., 2018). When experiencing stress or nutritional imbalance, stems can weaken and lose their ability to resist external forces, resulting in the leaning or even breakage of the entire or partial plant structure (Lv *et al*., 2023). The soybean gene *CS1*, implicated in lodging resistance and high‐density planting adaptability, regulates stem strength by modulating auxin transport and vascular bundle development (Xu *et al*., 2025). However, there remains a lack of comprehensive research on increasing lignin content to enhance stem strength and improve lodging resistance.

Here, in a mutant screen to identify genes contributing to soybean stem strength, we identified a mutant in the field named *lodging‐related mutant 3* (*lrm3*) that has weaker stem strength, less lignin and substantial yield penalties. *LRM3* encodes an E3 ubiquitin ligase containing a U‐box domain, which regulates stem strength by physically interacting with MYB6 and promoting its ubiquitination‐mediated degradation via the 26S proteasome. MYB6 binds to the promoter regions of *PHENYLALANINE AMMONIA LYASE 1* and *2* (*PAL1*, *2*) and represses their expression, downregulating lignin biosynthesis and thereby reducing stem strength. These findings expand our understanding of E3 ligase‐mediated regulation of lignin biosynthesis, stem strength and wider developmental programs for soybean, while providing valuable insights for engineering and breeding strategies to mitigate stem lodging as the demand for plant protein and oils continues to grow.

## Results

### Lodging phenotype of *lrm3* arises from the loss‐of‐function of a U‐box protein

**Figure 1:**
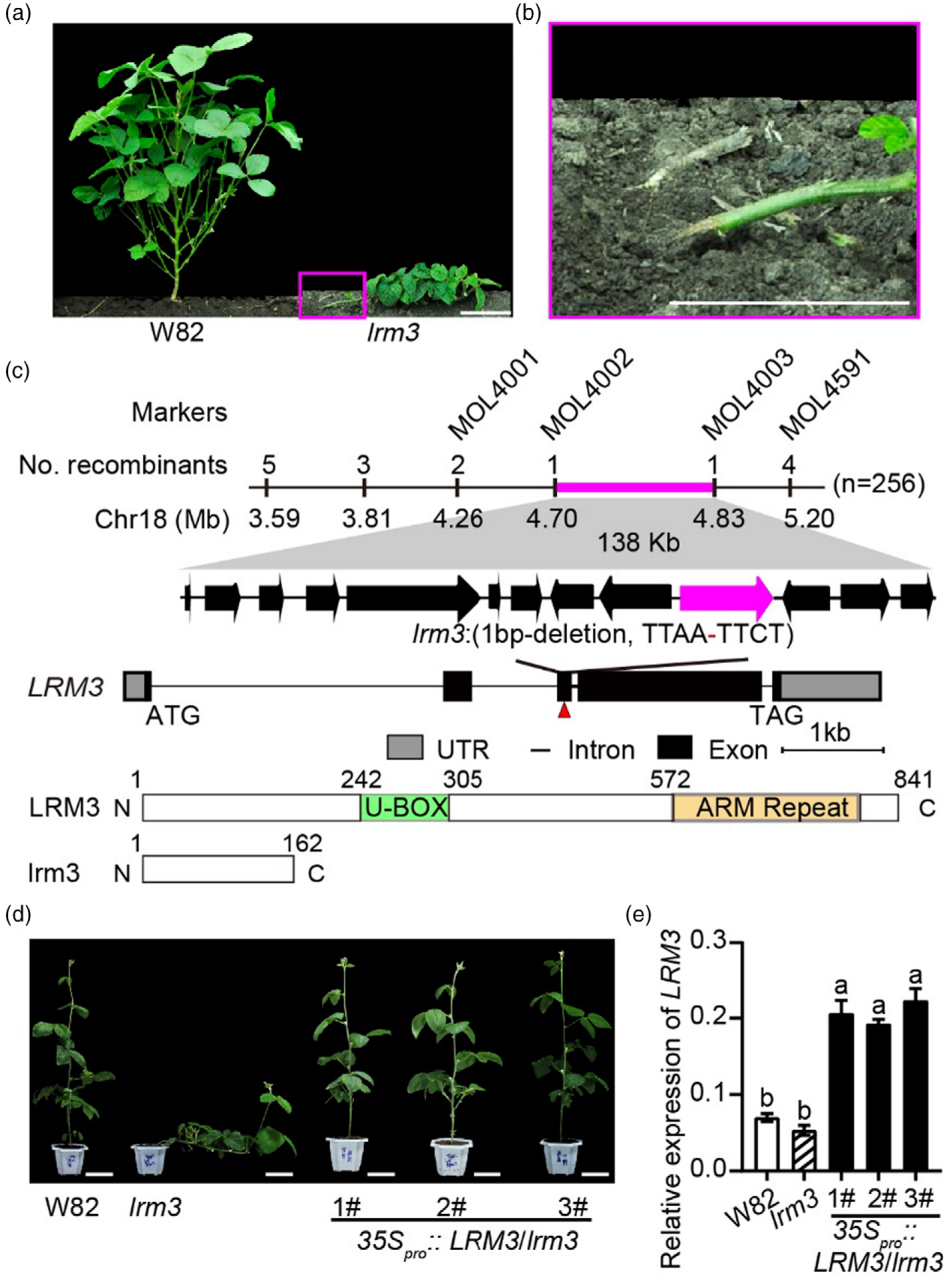
Map‐based cloning of the lodging‐prone soybean *LRM3* locus. (a, b) Representative images of field‐grown wild‐type W82 and *lrm3* knockout mutants at the V7 stage. Scale bar = 10 cm. Images within pink frames depict enlarged views of the stem region proximal to the ground. (c) Mapping of the *lrm3* candidate gene. Top: *LRM3* was fine‐mapped to a 138‐kb region (pink) between the markers MOL4002 and MOL4003 on chromosome 18. Middle: Genomic structure of *Glyma.18G055200*. The location of the mutation in *lrm3* is denoted by the red triangle. Bottom: Domain representations of the wild‐type *Glyma.18G055200* and mutant lrm3 protein. lrm3 is truncated to 162 amino acids due to a stop codon resulting from a base substitution in the *lrm3* mutant. (d) Photos of wild‐type W82, *lrm3* mutant and *lrm3* complementary transgenic plants at the V7 stage grown in the greenhouse. Scale bar = 10 cm. (e) The expression of the *LRM3* gene relative to *CONS4* of W 82, *lrm3* mutant and *lrm3* complementary transgenic plants. Relative expression data are presented as mean ± standard deviation (*n* = 3).

To dissect the regulatory mechanisms underlying lodging resistance, we isolated a lodging‐prone mutant designated as *lodging‐related mutant 3* (*lrm3*) from a population generated by ethyl methane sulfonate (EMS) mutagenesis of Williams 82 (W82) (Gao *et al*., 2017). Compared to W82, the *lrm3* mutant displayed a lodging phenotype following the emergence of the seventh trifoliate leaf (the V7 stage), attributable to inadequate stem strength to support the plant's weight (Figures 1a,b and S1A–C). Furthermore, *lrm3* mutants showed a significant reduction in both pod number and yield per plant (Figure S1D,E).

To investigate the genetic basis of the *lrm3* mutant phenotype, we crossed *lrm3* mutants with a local cultivar, HeDou 12. In this F_2_ population, a total of 145 wild‐type and 44 mutant plants were observed, conforming to a Mendelian inheritance pattern with a 3:1 ratio (*x* ^2^ = 0.153, *df* = 1, *P* = 0.696 Table S1), indicating that the lodging phenotype of *lrm3* is caused by a single recessive locus. The *LRM3* locus was mapped to a physical interval of 3.5–5.5 Mb (2 Mb) on chromosome 18 through improved Bulk Segregant Analysis (Wu *et al*., 2023; Zhou *et al*., 2021) (Figure S2A,B). We further narrowed down the *LRM3* locus to a 138‐kb region between markers MOL4002 and MOL4003 using 256 F_2:3_ individuals (Figures S2C and 1c). Comparative sequencing of the candidate genomic region between the *lrm3* mutant and W82 revealed a single SNP in the exon of *Glyma.18G055200* among the 13 annotated genes within this interval. This mutation introduces a premature stop codon, resulting in the truncation of the encoded protein (Figures 1c, S3 and S4C).

*Glyma.18G055200* contains five exons and four introns and encodes a protein comprising 841 amino acids, which includes a U‐box domain and six ARMADILLO repeat units (Figure 1c). Phylogenetic analysis demonstrated that it is the homologous gene of PUB4 (Plant U‐box‐4), an E3 ubiquitin ligase, in *Arabidopsis* (Figure S4). Quantitative PCR analysis demonstrated that *LRM3* is expressed in all tissues, predominantly in leaves (Figure S5A). Subcellular localization studies in tobacco leaf epidermal cells suggested that LRM3 is likely localized to both the plasma membrane and the nucleus (Figure S6).

To confirm that the loss of function of *Glyma.18G055200* causes the lodging phenotype in the *lrm3* mutant, we constructed an overexpression vector containing the coding sequences of *LRM3* and introduced it into the *lrm3* mutant. All six independent transformants displayed the wild‐type phenotype, and three independent complemented transgenic lines were selected for further detailed characterization. Genetic analysis also confirmed that the coding sequences of *LRM3* have been successfully introduced in these three lines under the *lrm3* mutant background, as evidenced by the detection of exogenous vector and LRM3‐Flag protein expression results (Figure S7A,B). Quantitative PCR (qPCR) analysis was performed to detect the expression level of *LRM3* in the complementary lines. These results demonstrated that ectopic expression of *LRM3* in the mutant was able to restore the lodging phenotype to the wild‐type state (Figure 1d,e).

**Figure 2:**
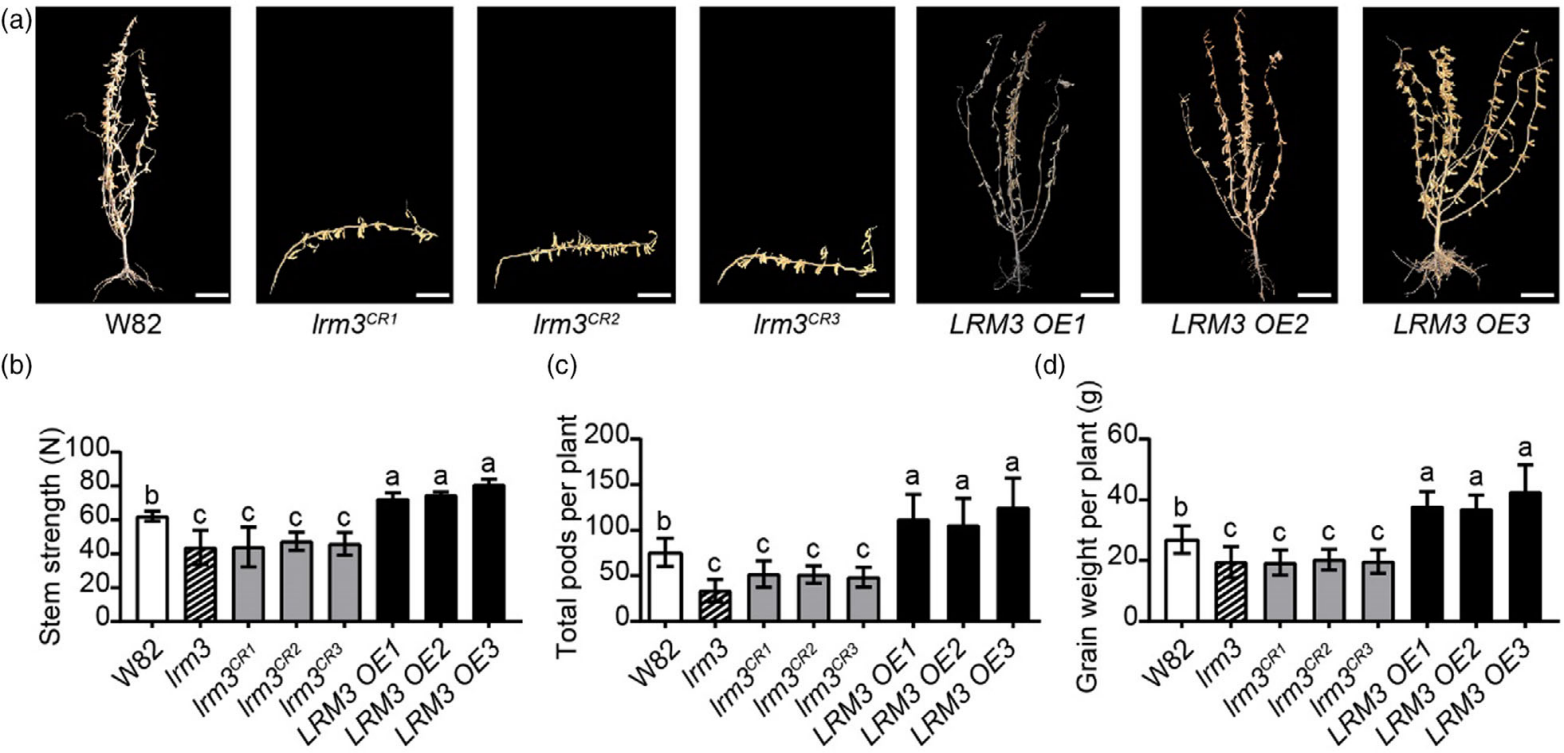
*LRM3* positively regulates stem strength of soybean. (a) Representative plant images of W82 (wild type), *lrm3* ^*CR*^ (CRISPR/Cas9 mutant) and *LRM3 OE* (overexpression) lines at the mature stage in the field. Scale bar = 10 cm. (b) Rind‐penetration strength at the first internode in W82, *lrm3* mutant, *lrm3* ^*CR*^ and *LRM3 OE* lines at the R1 stage. Stem strength data are presented as mean ± standard deviation (*n* = 5). Statistical comparison was performed by one‐way ANOVA at *P* < 0.05; different lowercase letters indicate significant differences. (c, d) Agronomic traits of W82, *lrm3* mutant, *lrm3* ^*CR*^ and *LRM3 OE* lines at the mature stage, pods per plant (C) and grain weight per plant (D). Traits data are presented as mean ± standard deviation (*n* = 30). Statistical comparison was performed by one‐way ANOVA at *P* < 0.05; different lowercase letters indicate significant differences.

We also knocked out *LRM3* using CRISPR/Cas9 in the W82 background. The resulting knocked‐out mutants (designated *lrm3* ^*CR1–3*^) encode truncated forms of *lrm3* (43, 44 and 42 amino acids in length, respectively) that lack the entire U‐box domain and ARM repeat domain (Figure S8A–E). All these knockout mutants exhibited reduced stem strength, similar to the EMS‐induced mutant *lrm3* (Figure 2a,b). In contrast, knocking out the *LRM3* homologous gene, *Glyma.11G161500*, did not produce any noticeable effect on stem strength (Figure S9A–D).

Additionally, stable transgenic lines with *LRM3* overexpression in W82 were also generated, and three lines were selected based on their significantly increased *LRM3* transcript levels in stems (*LRM3 OE 1–3*, Figures 2a and S10). Agronomic character data at the maturity stage showed the *LRM3* overexpression lines demonstrated a marked increase in both pod number and yield per plant. In contrast, the *lrm3* ^*CR1–3*^ knockout mutants exhibited a significant reduction in both pod number and yield (Figure 2c,d). These results suggest that LRM3 may enhance soybean yield by positively regulating stem strength.

### LRM3 regulates stem strength by affecting lignin synthesis

**Figure 3:**
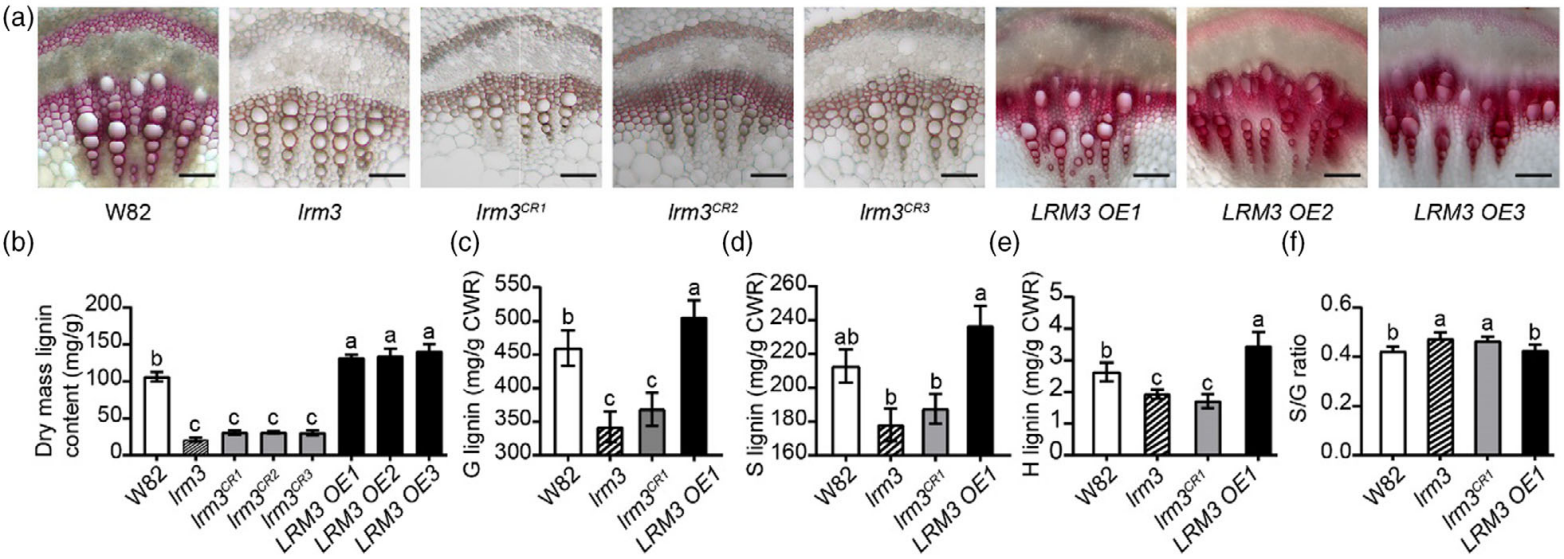
*LRM3* regulates stem strength by affecting lignin synthesis. (a) Cross‐sections of stems at the 6th internode of W82, *lrm3* mutant, *lrm3* ^*CR1‐3*^ and *LRM3 OE1‐3* at the V7 stage stained with phloroglucinol‐HCl, respectively. Scale bar = 100 μm. (b) Lignin contents at the first internode in W82, *lrm3* mutant, *lrm3* ^*CR1*^ and *LRM3 OE1* at the V7 stage, which are presented as means ± standard deviation (*n* = 5). Statistical comparison was performed by one‐way ANOVA at *P* < 0.05; different lowercase letters indicate significant differences. (c–e) Quantification of thioacidolytic lignin monomers (c, G‐lignin content, d, S‐lignin, content, e, H‐lignin content) in the cell walls of W82, *lrm3* mutant, *lrm3* ^*CR1*^ and *LRM3 OE1* lines. CWR, cell wall residues. (f) The ratio of S lignin to G lignin content presented in (d) and (c). The lignin composition data are presented as mean ± standard deviation (*n* = 3). Statistical comparison was performed by one‐way ANOVA at *P* < 0.05; different lowercase letters indicate significant differences. CWR, cell wall residues.

To elucidate the role of the *LRM3* gene in regulating stem strength, we analysed the cell wall composition in stems. The results showed that lignin content was significantly reduced by approximately 70%–80% in both *lrm3* mutants and *lrm3* knockout lines compared to the wild type. In contrast, lignin content was substantially increased by 30% in *LRM3* overexpression lines (Figure 3a,b). Interestingly, no significant differences were observed in the levels of cellulose or hemicellulose between the mutants (including both *lrm3* and knockout lines) and the wild type (Figure S11A,B). Furthermore, phloroglucinol staining corroborated these findings, showing a marked reduction in lignin deposition in the mutants and enhanced lignin accumulation in the overexpression lines (Figure 3a,b).

The lignin composition was analysed in W82, *lrm3*, *lrm3* knockout mutants and *LRM3* overexpression plants by quantifying the content of lignin monomers. Compared to W82, G‐lignin content decreased by 117.41 mg/g (25.5%) in the *lrm3* mutants and by 91.04 mg/g (19.8%) in the *lrm3* knockout mutants. In contrast, G‐lignin content increased by 10% in the *LRM3* overexpression plants (Figure 3c). However, no significant differences were observed in S‐lignin content across these materials (Figure 3d,e). Further analysis revealed a significant increase in the S/G lignin ratio in both the *lrm3* mutants and the *lrm3* knockout mutants compared to W82, while no significant changes were noted between the *LRM3* overexpression plants and W82 (Figure 3f). This result demonstrates that the *lrm3* mutation leads to a reduction in the levels of G‐lignin and H‐lignin, as well as alters the S/G lignin ratio.

### Degradation of MYB6 mediated by LRM3 through the 26S proteasome pathway

**Figure 4:**
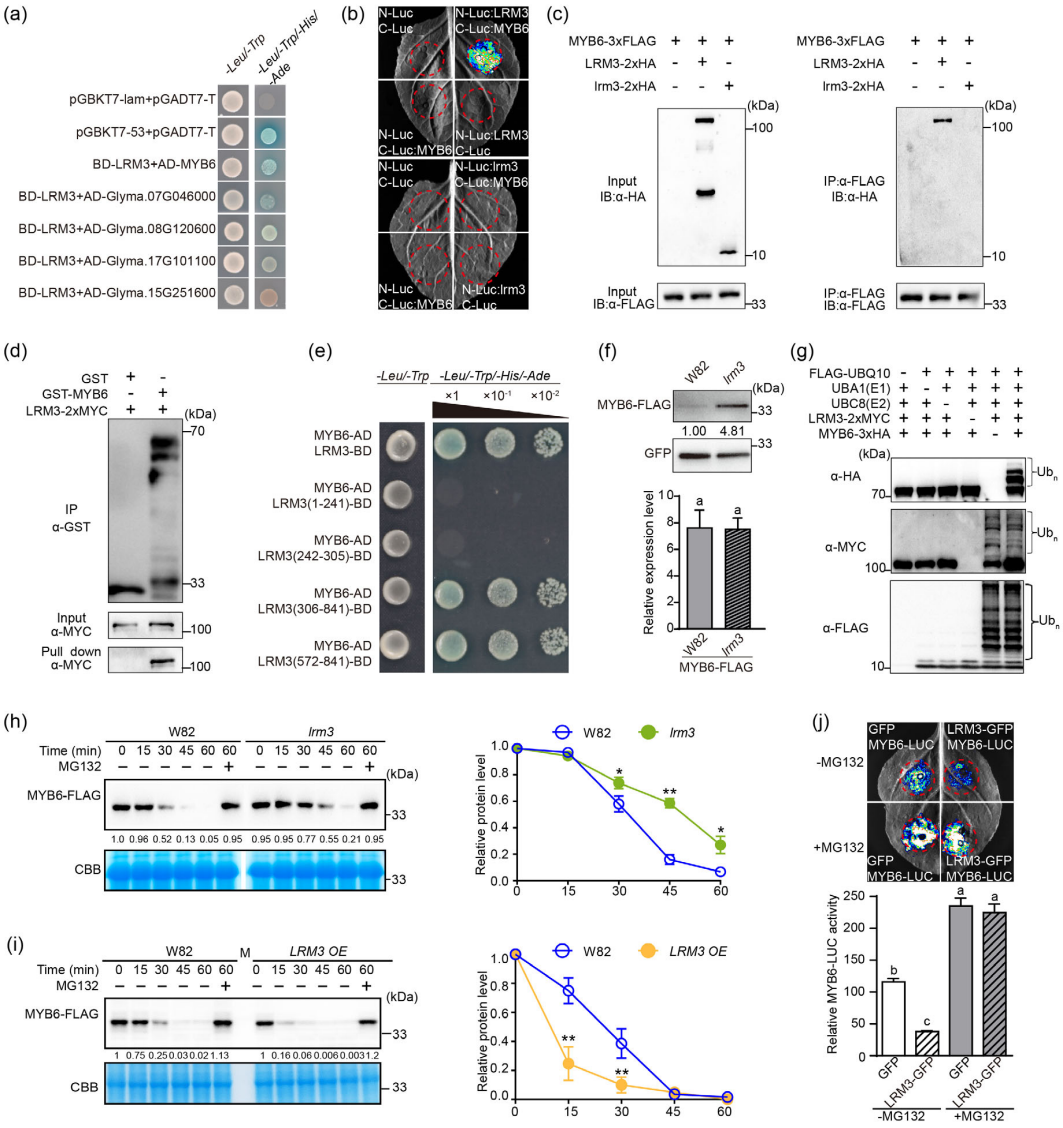
LRM3 physically interacts with MYB6 and degrades MYB6 by ubiquitination via the 26S proteasome pathway. (a) Proteins interacting with LRM3 in a yeast two‐hybrid screen. (b) Split‐luciferase complementation assay of the interaction *in vivo* between LRM3 and MYB6 in transiently transgenic *N. benthamiana* leaves. (c) Co‐IP assay of interactions *in vivo* between LRM3 and MYB6, lrm3 and MYB6. Proteins before (Input) and after incubation (IP) were detected with anti‐FLAG and anti‐HA antibodies. (d) Pull‐down assay of the interaction *in vitro* between LRM3 and MYB6. The input was immunoblotted using anti‐GST and anti‐MYC antibodies. GST, GST empty vector. (e) Yeast two‐hybrid assay of LRM3 interaction domains. The numbers above the images indicate two serial dilutions. BD, GAL4 DNA‐binding domain; AD, GAL4 activation domain. (f) Top: MYB6‐3 × FLAG levels were detected by western blotting using anti‐FLAG antibodies. Bottom: Transcript levels of *MYB6*‐*3 × FLAG* in transgenic hairy roots generated in wild‐type and *lrm3* mutant backgrounds, corresponding to (top). One cassette contained *35S*_*pro*_:*GFP* as an internal control, and the other cassette carried a *35S*_*pro*_:*MYB6*‐*3 × FLAG* cassette for comparison of abundance between W82 and *lrm3*. Student's *t*‐test (two‐sided) was used to generate the *P* values. (g) MYB6 is ubiquitinated by LRM3. LRM3‐Myc activities were analysed by western blotting with an anti‐MYC antibody; an anti‐FLAG antibody was used to detect 6 × His‐FLAG‐AtUBQ10 Ub conjugates. (h, i) Cell‐free degradation assay showing LRM3 promotes the degradation of MYB6‐3 × FLAG. Recombinant MYB6‐3 × FLAG was incubated with equal quantities of total proteins extracted from wild‐type and *lrm3* (h) and LRM3_OE (i) leaves ± MG132. MYB6‐3 × FLAG was detected with anti‐FLAG antibodies, and numbers beneath each sample in (Left) correspond to relative protein levels. Coomassie Brilliant Blue (CBB) was used as a loading control. Right: Quantification of relative changes in MYB6‐3 × FLAG intensity determined by relative protein levels over time as shown in (Left). Error bars depict the mean ± standard error of *n* = 5. (j) MYB6‐LUC degradation by LRM3‐GFP. MYB6‐LUC was transiently expressed in *N. benthamiana* leaves with an empty‐vector control or LRM3‐GFP. Five individual leaves for each of the reporter and effector combinations were imaged and quantified. Error bars depict the mean ± standard error of *n* = 5. Different lowercase letters indicate statistically significant differences at *P* < 0.01 by one‐way ANOVA.

The potential interacting proteins of LRM3 were investigated using yeast two‐hybrid (Y2H) screening on a cDNA library generated from soybean seedlings (Table S3). Among the five candidates (Figure 4a), MYB6, the soybean homologue of the poplar R2–R3 MYB transcription factor LTF1, was found to negatively affect lignin biosynthesis and stem strength (Gui *et al*., 2019). Therefore, we selected MYB6 as the candidate interacting protein of LRM3 for further functional studies. Quantitative PCR analysis revealed that *MYB6* is expressed in all tissues, with the highest expression levels observed in leaves (Figure S12A). By transiently expressing MYB6‐GFP in *N. benthamiana* leaves, we observed exclusive GFP fluorescence localized to the nucleus (Figure S12B).

Luciferase complementation imaging (LCI) assays demonstrated that co‐expression of LRM3‐nLuc with cLUC‐MYB6 resulted in a strong LUC signal, while no signals were detected from lrm3‐nLuc and cLUC‐MYB6 co‐expression (Figure 4b). Co‐immunoprecipitation (Co‐IP) assays showed that only LRM3 (~110 kDa band), but not lrm3 (~15 kDa band), was co‐purified with MYB6, validating the interaction *in planta* (Figure 4c). Pull‐down assays also indicated that GST‐MYB6 could be pulled down by LRM3‐2 × MYC, demonstrating a direct interaction between LRM3 and MYB6 (Figure 4d). A protein‐deletion experiment revealed that the C‐terminal 306–841 amino acids of LRM3 are essential for interacting with MYB6 (Figures 4e and S13A). Expression in transgenic soybean hairy‐root lines showed that the accumulation of MYB6‐3 × FLAG in *Irm3* was higher than in the W82 background (Figure 4f), implying that loss of *LRM3* leads to increased accumulation of MYB6, independent of transcript levels.

The ubiquitination assay conducted in *Escherichia coli* revealed that LRM3‐MYC can facilitate the ubiquitination of MYB6‐3 × FLAG in the presence of E1 and E2 enzymes, while simultaneously undergoing self‐ubiquitination (Figure 4g). In a cell‐free degradation assay, MYB6‐3 × FLAG degraded more slowly in lrm3 protein extracts compared to W82 (Figure 4h), but faster in *LRM3 OE* extracts (Figure 4i). This degradation was further inhibited by the 26S proteasome inhibitor MG132 in both backgrounds (Figure 4h,i). The signals of luciferase fused with MYB6 in *N. benthamiana* leaves were attenuated upon co‐expression of LRM3‐GFP, while the destabilizing effects on MYB6 abundance were counteracted by MG132 (Figures 4j and S14A). These findings provide supporting evidence for the hypothesis that LRM3 promotes the degradation of MYB6 through the 26S proteasome.

**Figure 5:**
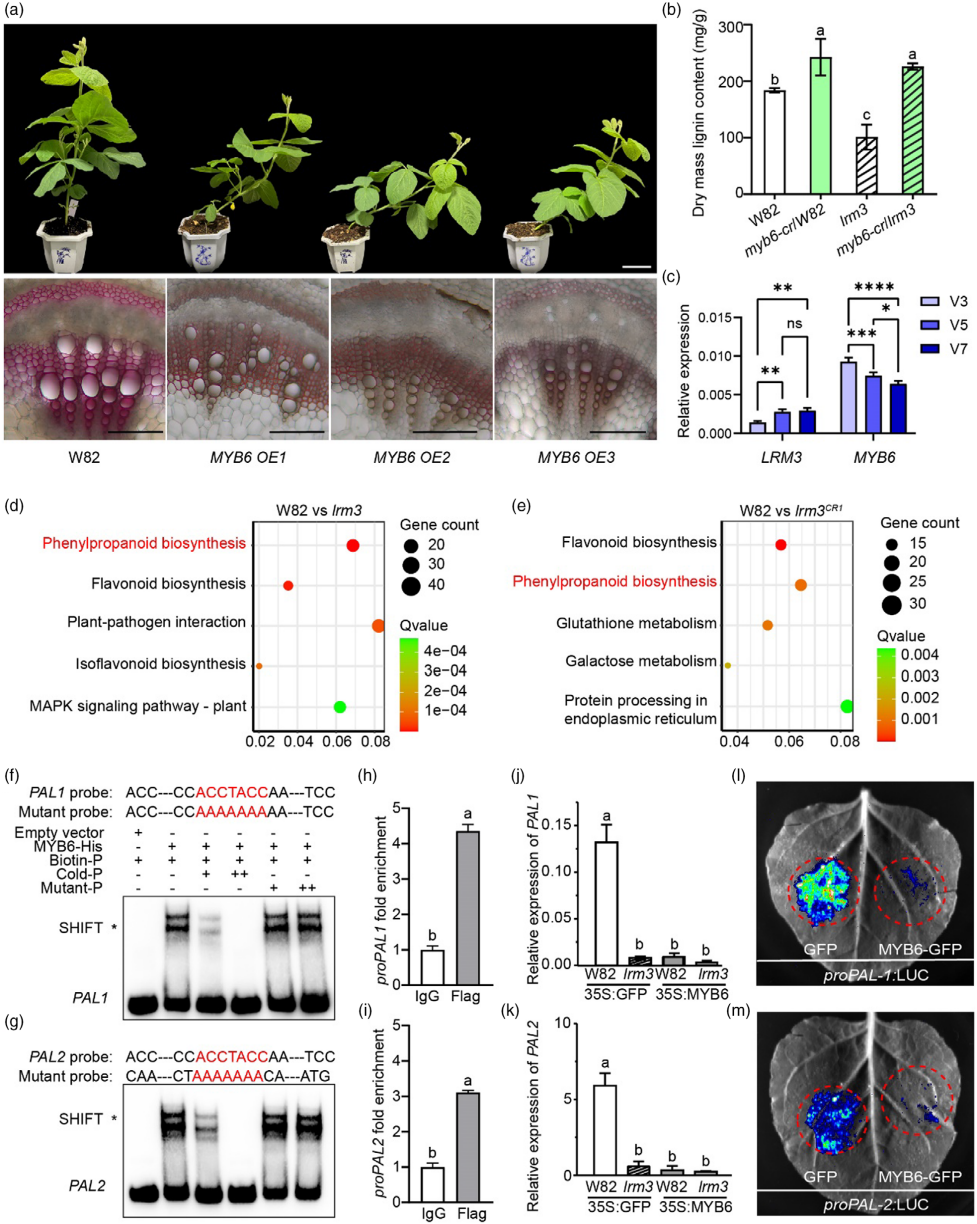
MYB6 binds to and represses the expression of *PALs* to downregulate lignin biosynthesis and influence soybean lodging. (a) Top: Photos of wild‐type W82 and stable‐transgenic *MYB6* overexpression plants at the V7 stage grown in the greenhouse. Scale bar = 10 cm. Bottom: Cross‐sections of stems at the 6th internode of Wild‐type and *MYB6* overexpression lines at the V7 stage stained with phloroglucinol‐HCl, respectively. Scale bar = 200 μm. (b) Lignin content in the transformed hairy roots of W82, *myb6‐cr*, *lrm3* and *myb6‐cr*/*lrm3* double mutant. Lignin content data are presented as mean ± standard deviation (*n* = 5). Statistical comparison was performed by one‐way ANOVA at *P* < 0.05; different lowercase letters indicate significant differences. (c) *LRM3* and *MYB6* expression in the fourth internode of Williams 82 at the V3, V5 and V7 stages. Relative expression data are presented as mean ± standard deviation (*n* = 3). Statistical comparison was performed by two‐way ANOVA (*P < 0.05, **P < 0.01, ***P < 0.001, ****P < 0.0001). (d, e) KEGG enrichment analysis of DEGs in RNA samples isolated from culms of *lrm3* (D) and *lrm3* ^*CR1*^ (e) compared to W82. The top‐five KEGG pathway terms are shown. Sphere size is proportional to the gene count, whereas the hue of the sphere is indicative of the *P* value. Three biological replicates were used for each genotype. (f, g) Electrophoretic mobility shift assay of *in vitro* binding between MYB6‐6 × His and its binding site in the *PAL1* (f) and *PAL2* (g) promoters. Probe sequences are as indicated, with the wild‐type and mutant binding motifs shown in red. P: probe. (h, i) CUT&Tag‐qPCR analysis of binding affinity to *PAL1* (h) and *PAL2* (i) promoter. After normalization with *PAL1* and *PAL2*, the relative enrichment in IgG was designed as 1 to normalize that in chromatin immunoprecipitated with anti‐Flag antibody. Error bars depict the mean ± standard error of *n* = 3. (j, k) qRT‐PCR validation of *PAL1* and *PAL2* expression in transgenic soybean hairy roots in the wild‐type W82 and *lrm3* mutant backgrounds. Relative expression data are presented as mean ± standard deviation, and relative expression was normalized to *CONS4*. Different lowercase letters indicate statistically significant differences at *P* < 0.01 by one‐way ANOVA. (l, m) MYB6‐GFP suppresses *PAL* promoter activity *in vivo*. In L and M, the indicated constructs were transiently co‐expressed in *N. benthamiana* leaves for 2 days. Luciferin was infiltrated 10 min before chemiluminescence imaging.

We constructed an overexpression vector carrying the coding sequences of *MYB6* and successfully introduced it into W82. Three independent transgenic lines were selected for comprehensive characterization. Notably, these transgenic plants exhibited a lodging phenotype that was similar to that observed in the *lrm3* mutant (Figure 5a). To verify the successful integration and expression of the *MYB6* gene in these transgenic plants, we detected the expression of the exogenous MYB6 protein and obtained positive results in PCR tests. These findings confirm the successful transgenic events in the three T_2_ generation transgenic plants (Figure S15A,B).

We also knocked out *MYB6* by root hair transformation in the W82 and *lrm3* backgrounds. Compared to the *lrm3* single mutant, the lignin content in the transformed roots of the *myb6* single mutant and *lrm3 myb6* double mutant is increased by more than 2 times (Figure 5b). This suggests that *MYB6* acts downstream of *LRM3* and is regulated by *LRM3*. To investigate the temporal and spatial expression patterns of *LRM3* and *MYB6* throughout the stem lignification process, we analysed their expression levels in the fourth internode during the V3, V5 and V7 stages.

Our results indicated that *LRM3* expression gradually increased from the V3 to V7 stages, suggesting its role in promoting lignification. In contrast, *MYB6* expression decreased significantly from the V3 to V7 stages and was lower in stems with high lignification (Figure 5c). These findings suggest there is a negative correlation between *LRM3* and *MYB6* expression levels during the stem lignification process.

### MYB6 represses *PAL* genes to negatively regulate lignin biosynthesis

To characterize the regulatory network involved in LRM3 function related to stem strength, we compared transcriptomes using samples from 2‐week‐old stems from W82, *lrm3* and *lrm3* ^*CR1*^. We identified a total of 3124 differentially expressed genes (DEGs) between W82 and *lrm3* (Figure S16A), as well as 2048 DEGs between W82 and *lrm3* ^*CR1*^ (Figure S16B). Among the overlapping 1533 DEGs (Figure S16C), we found significant enrichment for genes associated with the following processes: ‘cellular carbohydrate metabolic process’, ‘cellular polysaccharide metabolic process’, ‘glucan metabolic process’, ‘cellular glucan metabolic process’ and ‘polysaccharide metabolic process’ (Figure S16D,E). The phenylpropanoid biosynthesis pathway was statistically significantly enriched in KEGG analyses (Figure 5d,e). Within the DEGs, several key players in lignin biosynthesis were identified, including two *Phenylalanine ammonia lyases* (*PALs*), three *Cinnamate 4‐hydroxylases* (*C4Hs*), four *4‐coumarate: CoA Ligase* (*4CLs*), one each of *Catechol‐O‐methyltransferase* (*COMT*), *Cinnamoyl‐CoA reductase* (*CCR*) and *Cinnamyl alcohol dehydrogenase* (*CAD*), as well as four peroxidases (*POXs*) (Figure S17A). The expression of these genes in stem samples was confirmed to be downregulated in both *lrm3* and *lrm3* ^*CR1*^ compared to W82, as shown by RT‐qPCR (Figure S17B).

PAL catalyses the initial committed step in phenylpropanoid biosynthesis. Promoter sequence analysis identified the ACCTAC MYB‐binding motif (Zhong and Ye, 2012) in two soybean *PAL* genes: *Glyma.10G058200* (*PAL1*) and *Glyma.03G181600* (*PAL2*). EMSA experiments validated direct binding of MYB6 to these promoter regions *in vitro* (Figure 5f,g), confirming specific interaction between MYB6‐6 × His fusion protein and the ACCTAC elements in *PAL1*/*PAL2* promoters. CUT&Tag assays further revealed statistically significant 4‐ and 3‐fold enrichment of target sequences containing predicted MYB6 binding sites compared to negative controls in *PAL1* and *PAL2* promoters, respectively (Figure 5h,i).

Overexpression of MYB6‐GFP under the *CaMV 35S* promoter (*35S::MYB6‐GFP*) in hairy roots of wild‐type W82 soybean significantly repressed *PAL1* and *PAL2* transcript levels, whereas no such effect was observed in the *lrm3* mutant background (Figures 5j,k and S18). Conversely, *LRM3* overexpressing lines displayed marked upregulation of both *PAL* genes (Figure S19A,B). To directly demonstrate MYB6‐mediated repression, we constructed *PAL1*/*PAL2* promoter‐driven LUC reporter constructs and transiently co‐expressed them with MYB6 in *Nicotiana benthamiana* leaves. Co‐expression of MYB6 significantly suppressed luciferase activity driven by both *PAL* promoters (Figure 5l,m). As a control, western blot analysis confirmed comparable expression levels of GFP and MYB6‐GFP fusion protein under identical experimental conditions (Figure S20A,B). Collectively, these results establish that MYB6 directly represses *PAL1* and *PAL2* expression *in planta* (Figure S20C).

**Figure 6:**
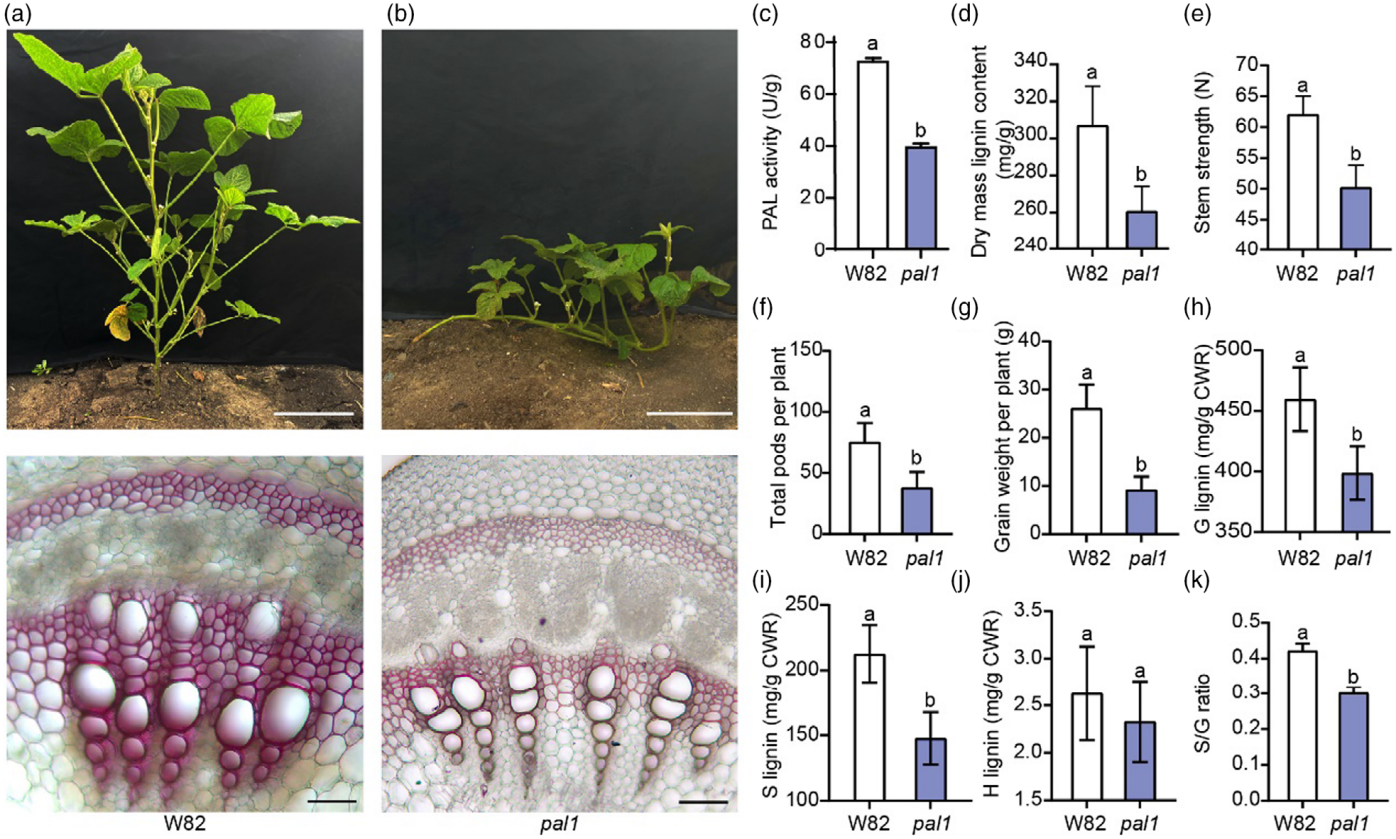
The *pal1* mutant negatively regulates lignin synthesis and stem strength. (a, b) Top: Phenotypes of wild‐type (a) and *pal1* mutant (b) plants. Scale bar = 10 cm. Bottom: Cross‐sections of stems of wild‐type (a) and *pal1* mutant (b) plants from the 6th internode at the V7 stage stained with phloroglucinol‐HCl. Scale bars = 50 μm. (c) PAL enzymatic activity in W82 and *pal1* mutant stems, which are presented as means ± standard deviation (*n* = 3). (d) Lignin content at the first internode in W82 and *pal1* mutant at the R1 stage, which are presented as means ± standard deviation (*n* = 3). (e) Rind‐penetration strength at the first internode in W82 and *pal1* mutant at the R1 stage, which are presented as means ± standard deviation (*n* = 5). (f–g) Agronomic traits at the mature stage for the indicated lines are shown in panel a in W82 and *pal1* mutant. Pods per plant (f) and Grain weight per plant (g). Traits data are presented as mean ± standard deviation (*n* = 30). (h–j) Quantification of thioacidolytic lignin monomers (c, g lignin content, d, s lignin, content, e, h lignin content) in the cell walls of W82 and *pal1* mutant. CWR, cell wall residues. (k) Ratio of S lignin to G lignin content presented in (i) and (h). The lignin composition data are presented as mean ± standard deviation (*n* = 3). Statistical comparison was performed by Student's *t*‐test (two‐sided) at *P* < 0.05; different lowercase letters indicate statistically significant differences at *P* < 0.05. CWR, cell wall residues.

To further validate the causal relationship between *PAL* gene inhibition and the lodging phenotype observed in *lrm3* mutants, we isolated an EMS‐induced *pal1* mutant harbouring a T → A transition in exon 2 of *Glyma.10G058200* (*PAL1*), resulting in a Thr‐to‐Ser substitution. The *pal1* mutant displayed a lodging phenotype comparable to *lrm3* (Figure 6a,b), confirming that *PAL1* disruption directly causes lodging in soybean. Biochemical analysis revealed significantly reduced PAL enzymatic activity in *pal1* compared to W82 (Figure 6c), accompanied by decreased stem mechanical strength and lignin content similar to *lrm3* (Figure 6d,e). Agronomic trait evaluation at maturity showed significant reductions in both pod number and grain weight per plant in *pal1* mutants (Figure 6f,g). Quantitative analysis of lignin monomer composition revealed that *pal1* mutants exhibited marked decreases in G‐lignin and S‐lignin contents, as well as the S/G ratio, relative to W82, while H‐lignin levels remained unchanged (Figure 6h–k). Collectively, these data establish a regulatory axis where LRM3 negatively modulates MYB6 activity to promote *PAL1*/*PAL2* expression and lignin biosynthesis, thereby maintaining stem structural integrity and yield potential.

### *LRM3* was selected during soybean domestication and modern breeding

**Figure 7:**
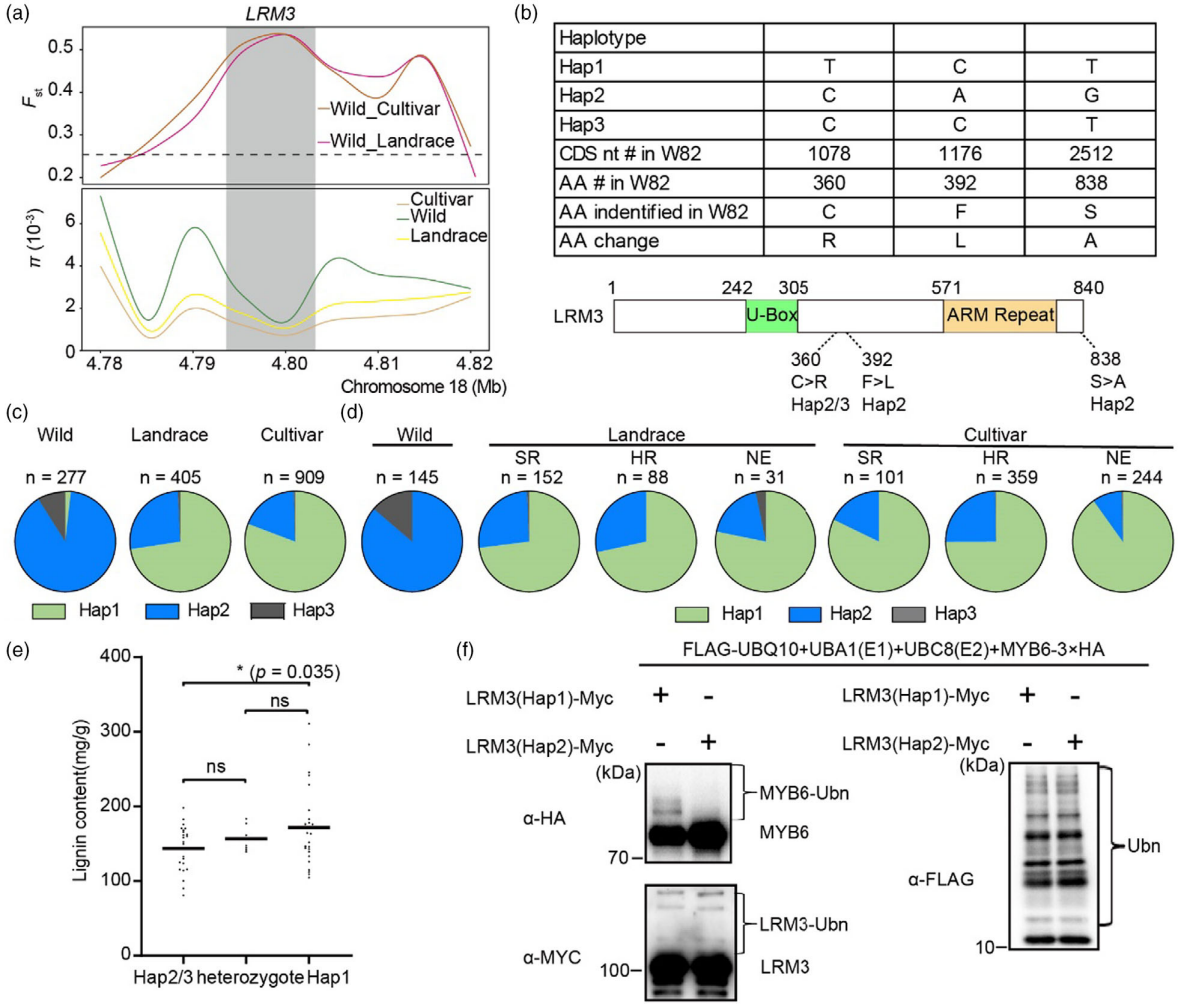
Haplotype analysis of *LRM3* in diverse soybean accessions. (a) Comparison of the ratio of *F*_ST_ and nucleotide diversity (π) over a ~40 kb genomic region containing the *LRM3* locus between wild and cultivated soybean germplasm. (b) *LRM3* haplotypes in natural populations. Top: Haplotypes of *LRM3*; Bottom: nonsynonymous mutant alleles of *LRM3*. (c) Pie charts of haplotype distribution in wild soybeans, landraces and improved cultivars. (d) Allelic distributions of Hap1/2/3 in subsets of Chinese landraces and cultivars (from c) according to region of origin. NE, northeast region of China; HR, Huanghuai region of China; SR, southern region of China. (e) Lignin content of *LRM3* ^*Hap1*^, *LRM3* ^*Hap2/3*^ heterozygous for *LRM3* ^*Hap1*^ and *LRM3* ^*Hap2/3*^ from the recombinant inbred lines. A one‐way ANOVA was used to generate the *P* values. *P < 0.05, ns, nonsignificant. Lignin content data are presented as mean ± standard deviation (*n* = 70). (f) Ubiquitination activity of different haplotypes. Lysates from *E. coli* strains expressing AtUBA1‐S, MBP‐MYB6‐3 × HA, AtUBC8‐S, 6 × His‐FLAG‐AtUBQ10 and LRM3(Hap2/3)‐MYC or LRM3(Hap1)‐MYC were analysed by western blotting with an anti‐HA antibody to detect MBP‐MYB6‐2 × HA MYB6 ubiquitination. LRM3‐MYC activities were analysed by western blotting with an anti‐MYC antibody; an anti‐FLAG antibody was used to detect 6 × His‐FLAG‐AtUBQ10 Ub conjugates.

To characterize the evolutionary trajectory of *LRM3* during soybean domestication, we performed genome‐wide scans for selective signatures in the *LRM3* promoter and flanking genomic regions using pairwise fixation index (*F*_ST_) and nucleotide diversity (*π*) analyses across 1591 soybean accessions representing wild (*Glycine soja*), landrace and improved cultivar groups (Shen *et al*., 2018; Zhou *et al*., 2016). Both population genetic metrics consistently identified a strong selective sweep spanning a 40‐kb interval harbouring the *LRM3* locus (Figure 7a). Phylogenetic analysis of this region revealed three major haplotypes (Hap1‐3), with Hap1 predominating in cultivated soybeans, while Hap2 and Hap3 were exclusive to wild *G. soja* accessions (Figures 7b and S21A). Hap2 and Hap3 displayed close genetic relatedness, suggesting Hap2 represents the ancestral haplotype from which other variants diverged. Notably, Hap2 also showed genetic proximity to landrace and cultivar groups (Figure S21B). Frequency analysis revealed a significant decline in Hap2/Hap3 frequencies from wild to landrace/cultivated groups, concurrent with a progressive increase in Hap1 prevalence during domestication (Figure 7c), indicating strong artificial selection favouring Hap1.

Subsequent characterization of allelic distribution patterns within a geographically stratified subset of 1120 Chinese soybean accessions (Figure 7d) revealed significant enrichment of the Hap1 allele in both landrace and cultivar groups across all analysed regions. This enrichment exhibited a pronounced north–south cline, with Hap1 frequencies highest in northeastern China and progressively lower in Huanghuai and southern regions. Comparative sequence analysis of the three haplotypes identified a nonsynonymous substitution at nucleotide position 1078 (C → T transition), resulting in an arginine‐to‐cysteine amino acid change (R → C) that discriminates wild soybean (*Glycine soja*) from cultivated genotypes (Figure 7b). This substitution is particularly intriguing due to the potential role of cysteine residues as ubiquitination sites, suggesting possible posttranslational regulation of LRM3 function.

To explore functional consequences of the identified Hap1 substitution, we analysed lignin content in a recombinant inbred line (RIL) population derived from a wild‐cultivated cross (*Glycine soja* × *Glycine max*). Genotyping revealed three distinct genotypes: wild soybean (C/C), heterozygotes (C/T) and cultivated lines (T/T). Consistent with genotype–phenotype association, C/C lines exhibited significantly lower lignin content compared to C/T heterozygotes, which in turn had lower levels than T/T cultivars (Figure 7e). We also assessed a *lrm3* mutant in which Cys360 was replaced with Arg (C360R), resulting in attenuated ubiquitination activity (Figure 7f). Collectively, these findings provide genetic and biochemical evidence that artificial selection for the Hap1 allele during domestication enhanced lignin biosynthesis and stem strength through modulation of ubiquitination‐dependent regulatory pathways.

## Discussion

Amidst the twin challenges of rapid global population growth and constrained arable land, enhancing crop yield potential through increased planting density has emerged as a pivotal strategy for ensuring food security. However, the heightened risk of lodging arising from dense cultivation presents a substantial threat to yield stability, thereby posing a core dilemma in modern crop breeding: balancing high productivity with lodging resistance (Tu *et al*., 2022). Historical milestones in lodging‐resistance breeding, such as the semi‐dwarf crop improvements during the ‘Green Revolution’, were achieved by modulating gibberellin (GA) signalling pathways through key genes like rice *SD1* and wheat *Rht‐1* (Li *et al*., 2018; Peng *et al*., 1999; Sasaki *et al*., 2002). The recent discovery of the *Rht‐1* haplotype in wheat – an innovation that integrates GA and brassinosteroid (BR) signalling to reconcile lodging resistance with nitrogen‐use efficiency (Song *et al*., 2023) – highlights the pressing need for mechanistic advancements in breeding programs aimed at enhancing stalk strength.

**Figure 8:**
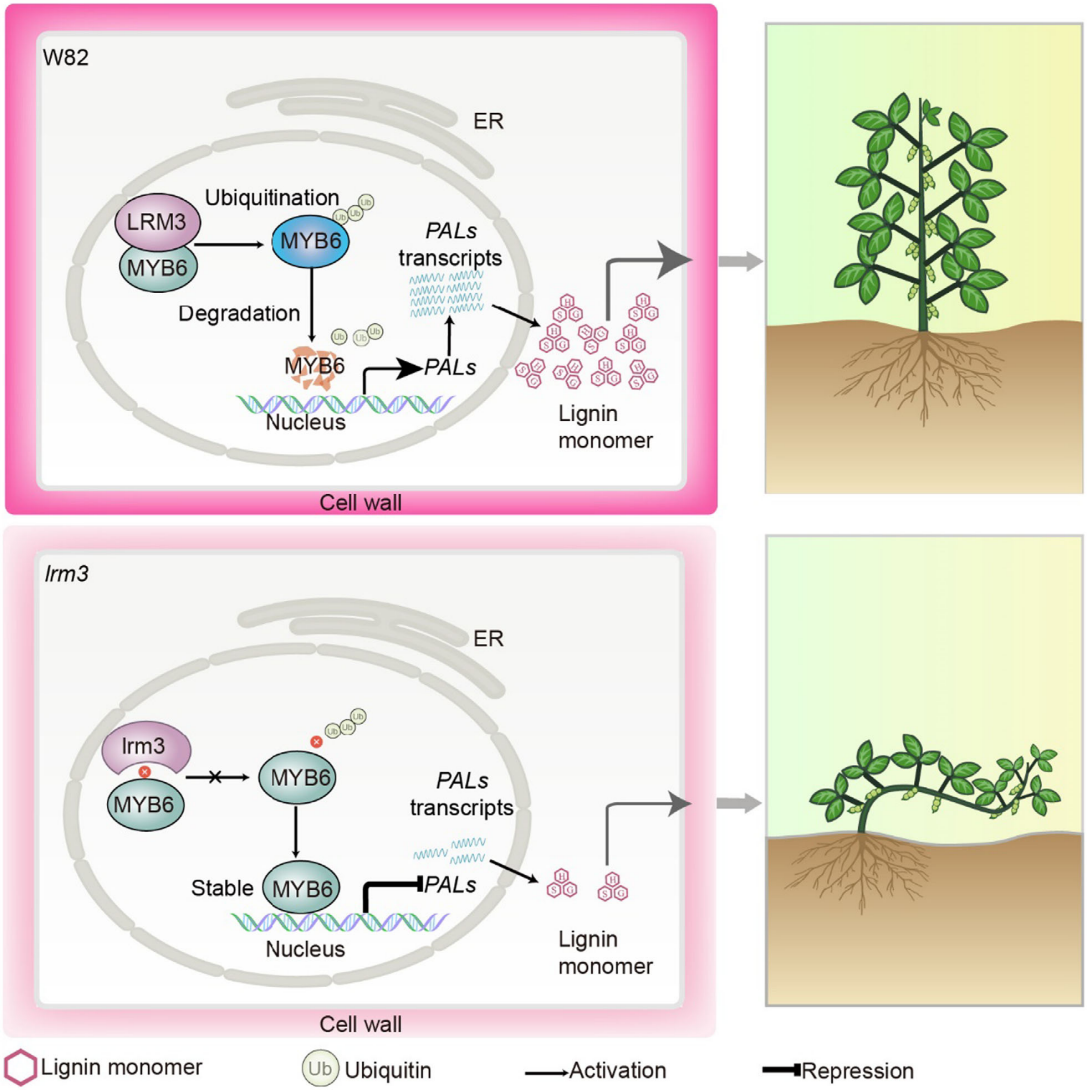
Model of soybean lignin‐biosynthesis regulation by LRM3. In the wild type (top), the E3 ligase LRM3 ubiquitinates the transcription factor MYB6, leading to its degradation via the 26S proteasome. This results in the induction of genes involved in lignin biosynthesis, including *PAL1 and PAL2*, via the phenylpropanoid pathway. In contrast, mutation of *lrm3* (bottom) leads to the overaccumulation of the repressor MYB6 and ongoing inhibition of downstream genes associated with lignin biosynthesis, ultimately causing insufficient lignin production, weaker cell walls in stems and soybean lodging.

As a member of the PUB E3 ubiquitin ligase family, LRM3 exhibits evolutionary divergence from *Arabidopsis* AtPUB4 – a gene implicated in tapetum development and pathogen defence (Desaki *et al*., 2019; Kinoshita *et al*., 2015; Wang *et al*., 2013, 2022; Yu *et al*., 2022) – underscoring a lineage‐specific adaptation in soybean for lignin biosynthesis regulation. Population genetic analysis of 1591 soybean accessions identified the *LRM3* Hap1 variant as a domestication‐associated allele significantly enriched in cultivated varieties and landraces, particularly in high‐latitude regions such as northeastern China (Figure 7). This geographic distribution pattern implies adaptive selection for improved stem strength under dense planting conditions, as Hap1 carriers exhibit superior yield performance and lodging resistance in high‐density field environments. Substantial advances have been made in deciphering the molecular mechanisms of soybean lodging resistance. For instance, *GmMYB14* modulates plant architecture through the activation of brassinosteroid (BR) signalling (Chen *et al*., 2021), *GmCRY1* alleviates low‐light‐induced etiolation by stabilizing DELLA proteins (Lyu *et al*., 2021), and *RIN1* controls internode elongation via a gibberellin (GA) metabolic regulatory module (Li *et al*., 2023). In contrast, our study uncovers a distinct mechanistic pathway: LRM3 enhances lodging resistance by precisely coordinating the spatiotemporal patterns of lignin deposition in the secondary cell walls of stem vascular tissues (Figure 8).

The ubiquitin–proteasome system (UPS) is central to regulating plant development and metabolism. E3 ubiquitin ligases, key components of this system, modulate lignin content by targeting enzymes or transcription factors in the phenylpropanoid pathway for ubiquitination and degradation. In *Arabidopsis*, Kelch domain‐containing F‐box proteins (KFBs), including KFB01, KFB20, KFB39, KFB50 and SMALL AND GLOSSY LEAVES1 (SAGL1), govern the stability of PAL proteins. These F‐box proteins facilitate PAL ubiquitination and proteasomal degradation, thereby reducing lignin biosynthesis (Yu *et al*., 2019; Zhang *et al*., 2013, 2017). Similarly, the bamboo F‐box protein PeKFB9 acts as a negative regulator of lignin biosynthesis by mediating the 26S proteasome‐dependent degradation of PePAL10, leading to reduced lignin accumulation (Yang *et al*., 2024).

Lignin biosynthesis is also governed by transcriptional regulatory networks of transcription factors, many of which serve as direct targets of the ubiquitin–proteasome system (UPS). For example, protein levels of VASCULAR‐RELATED NAC‐DOMAIN7 (VND7) – a key regulator of vessel differentiation in *Arabidopsis –* are tightly controlled via 26S proteasome‐mediated degradation (Phookaew *et al*., 2024; Yamaguchi *et al*., 2008). The E3 ligase SINAT5 (SEVEN IN ABSENTIA OF ARABIDOPSIS THALIANA 5) modulates plant lignin biosynthesis by interacting with the NAC transcription factor NAC1, promoting its ubiquitination and subsequent proteasomal degradation (Olsen *et al*., 2005). In cotton, the E3 ligase GhHUB2 (Histone H2B monoubiquitination) mediates polyubiquitination and degradation of GhKNL1 (a KNAT7 homologue), thereby relieving transcriptional repression of downstream genes involved in fibre elongation and secondary cell wall synthesis to facilitate fibre development (Feng *et al*., 2018).

MYB transcription factors, a large gene family in plants, play a central role in regulating lignin biosynthesis. In poplar, the E2 ubiquitin‐conjugating enzyme PtoUBC34 modulates lignin synthesis by interacting with transcriptional repressors PtoMYB156 and PtoMYB221, thereby regulating their protein stability (Zheng *et al*., 2019). In Chinese white pear, the RING‐H2 FINGER E3 ligase PbRHY1 promotes 26S proteasome‐dependent degradation of PbMYB80, relieving its repression of key lignin pathway genes and enhancing biosynthesis (Wang *et al*., 2024). Our study identifies a conserved posttranslational regulatory mechanism in soybean: the E3 ligase LRM3 promotes lignin accumulation by ubiquitinating and degrading the transcription factor MYB6, thereby alleviating its repression of *PAL1* and *PAL2* (Figure 8). This work provides the first evidence of a UPS‐mediated regulatory module for lignin metabolism in soybean, highlighting the evolutionary conservation of such ubiquitin‐dependent pathways across plant species.

By elucidating the LRM3‐mediated degradation of MYB6 to promote lignin biosynthesis, this study enhances the mechanistic understanding of soybean lodging resistance. Beyond soybean, it underscores the functional utility of ubiquitination regulatory modules in tailoring secondary cell wall properties to improve stalk strength – a strategy with broad translational potential across staple crop species.

## Materials and methods

### Plant materials and growth conditions

The soybean cultivars ‘Williams 82’ (W82) and ‘Hedou 12’ were obtained from the Chinese Academy of Agricultural Sciences. The *lrm3* mutant was generated in the W82 background by treatment of seeds with 0.5% v/v ethyl methane sulphonate (EMS) (Dai *et al*., 2018; Wang *et al*., 2020). To investigate the phenotypes of W82 and *lrm3* under natural conditions, plants were cultivated in the field in Changchun, Jilin province, China (43.05° N, 124.18° E). These plants were sown at the beginning of May and harvested in October 2020. Each row was 2 m long, with a spacing of 65 cm between rows. About 20 plants were sown in each row, and three rows were planted for each variety. Harvesting was performed in October 2020 or in a controlled‐climate chamber set to 28 °C with a relative humidity of 50% and a 14‐h photoperiod at 180 μmol photons/m^2^/s.

*Nicotiana benthamiana* was used for subcellular localization, luciferase‐complementation assays and degradation assays. Plants were cultivated in soil at 23 °C and 70% humidity with a 10‐h photoperiod at 100 μmol photons/m^2^/s.

### Evaluation of lignin, hemicellulose or cellulose contents

At the V7 stage, the first internodes were selected and dried at 80 °C for more than 3 days, and the lignin content was determined using the lignin content detection kit (BC4200, Solarbio, Beijing, China); hemicellulose or cellulose was determined using the hemicellulose or cellulose content detection kit (BC4280 and BC4440, Solarbio, Beijing, China) according to the manufacturer's instructions.

### Phenylalanine ammonia‐lyase activity assay

At the V7 stage, the eighth internodes were selected to detect the phenylalanine ammonia‐lyase (PAL) activity using the detection kit (BC0210, Solarbio, Beijing, China) according to the manufacturer's instructions.

### Lignin monomer measurement

To measure the monomeric composition of lignin, extractive‐free cell walls were prepared from stems at the V7 stage, with three biological replicates, each consisting of at least five plants. The monomeric composition was then determined using the thioacidolysis method and quantified via gas chromatography with flame ionization detection as previously described (Liu, 2012).

### Map‐based cloning of *LRM3*

To generate a mapping population for cloning *LRM3*, the *lrm3* mutant (W82 background) was crossed with Hedou 12 to produce an F_2_ population. The markers listed in Table S2 were used to map F_2_ plants expressing the *lrm3* mutant phenotype. For bulk‐segregant analysis, 40 F_2_ plants with the mutant phenotype were re‐sequenced using Illumina HiSeq2000 at a depth of approximately 30×. Genomic DNA was extracted from each individual plant and pooled equally to create separate wild‐type and mutant pools. Sequencing libraries were constructed for each pool as well as for the parental lines. Genomic DNA was sheared according to the Illumina TruSeq DNA PCR‐free prep kit protocol before library preparation. Reads from both bulk and parental lines were aligned to the updated W82 (Schmutz *et al*., 2010) reference genome using bwa software with default parameters, followed by SNP calling using GATK software. Candidate regions showing (ΔSNP index) > 0.5 were identified (McKenna *et al*., 2010) as previously reported (Wu *et al*., 2023; Zhou *et al*., 2021).

### Multiple‐sequence alignment and phylogenetic analysis

We searched for LRM3 homologues in Phytozome (PhytozomeV12.0;) in the genomes of *Glycine max*, *Phaseolus vulgaris*, *Medicago truncatula*, *Prunus persica*, *Vitis vinifera*, *Solanum tuberosum*, *Arabidopsis thaliana*, *Zea mays*, *Sorghum bicolor*, *Brachypodium distachyon*, *Panicum hallii*, *Oryza sativa*, *Ananas comosus*, *Marchantia polymorpha*, *Physcomitrium patens* and *Amborella trichopoda*. The database was queried using *Glyma.18G055200* with BLASTP (https://blast.ncbi.nlm.nih.gov/Blast.cgi). Alignment of the full‐length protein sequences was performed, and a phylogenetic tree was constructed using MEGA 7.0 software (Kumar *et al*., 2016).

### RNA isolation and quantitative real‐time PCR (qRT‐PCR)

The total RNA was extracted from roots, stems, leaves, unopened flowers, open flowers, pods and shoot apical meristems (SAM) using Trizol following the manufacturer's instructions (Invitrogen Life Technologies). Subsequently, the RNA was treated with RNase‐free DNase I (Cat. no. 2270; Takara) at 37 °C for 30 min. Reverse transcription was performed using the Prime Script RT‐PCR Kit (Cat. no. RR014; Takara) according to the manufacturer's protocol with 1 μg of RNA in a 20 μL reaction. qRT‐PCR analysis was conducted using SYBR premix Ex Taq (Cat. no. RR420; Takara) on the GeneAmp 5700 Sequence Detection System (Applied Biosystems; http://www.appliedbiosystems.com), as per the manufacturer's instructions. Three biological replicates with three technical replicates each were used for amplification. Relative expression levels were calculated using the 2^−∆∆Ct^ method after normalization to *CONS4* (ATP‐binding‐cassette transporter gene, *Glyma12G020500*) (Gao *et al*., 2021).

### CRISPR/Cas9 vector construction and soybean transformation

To generate *LRM3* knockout alleles, we used CRISPR/Cas9 gene editing (Chen *et al*., 2021). Single‐guide RNAs (sgRNAs) to target Cas9 were identified using CRISPR‐P version 2.0. A pair of 23‐bp long oligonucleotides (5′‐ ATTAAAGTATTACCAGAAGGCAG‐3′ and 5′‐AACCTGCCTTCTGGTAATACTTT‐3′), designed specifically for the *LRM3* sequence, were annealed and cloned into a modified VK005‐04‐soU6‐2‐Ubi3 expression vector (Du *et al*., 2016). The resulting recombinant plasmid (VK005‐LRM3) was introduced into *Agrobacterium tumefaciens* EHA105, which was subsequently used to transform W82 cotyledon explants (Zhao *et al*., 2016). To detect CRISPR‐Cas9‐induced transgenic lines in T_0_ plants, leaves were placed in 1.5 mL centrifuge tubes and crushed with a pestle. Detection buffer (200 μL) was added and mixed with each sample. PAT/BAR test strips (Youlong Biotech Co., Ltd, China) were inserted into the above centrifuge tubes, and the positive red bands appeared in the strips after 10 min. To identify the genotypes of edited plants, genomic DNA was extracted from the seedlings of wild‐type and all positive transgenic plants and target sites were amplified by PCR. The edited mutations were confirmed by DNA sequencing. Three independent transgenic lines with *LRM3* knocked‐out were obtained for subsequent phenotypic analysis.

### Histochemical staining

The 6th‐internode stems from V7 stage W82 and *lrm3* mutants were manually sectioned and subjected to staining with a solution containing 0.5% (w/v) phloroglucinol in 12% HCl as previously reported (Gui *et al*., 2019). Samples were examined under a light microscope (DM2500, Leica, Germany).

### Subcellular localization of LRM3 and MYB6

The coding sequence of *LRM3* and *MYB6* without the translational stop codon was amplified from cDNA, cloned into the *pCAMBIA1300‐35S‐GFP‐RBS* vector (Wang *et al*., 2020) in‐frame with C‐terminal GFP and transformed into *A. tumefaciens* EHA105 for transient expression in *N. benthamiana* leaves. Infiltrated leaves were cultivated for 2 days before imaging. Subcellular localization of LRM3‐GFP was monitored by visualizing GFP fluorescence using a Nikon confocal microscope C2 (Japan) with excitation at 488 nm and emission at 495–540 nm (Liang *et al*., 2022).

### Protein‐interaction assays

The full‐length *LRM3* coding sequence, fused to the GAL4 DNA‐binding domain in the *pGBKT7* vector (Clontech), was employed as bait in a yeast two‐hybrid assay screen of a soybean cDNA library conducted in our laboratory. Yeast AH109 cells harbouring the bait vector BD‐LRM3 were subsequently transformed with the soybean cDNA library cloned into the prey vector *pGADT7* (Clontech) and selected on yeast synthetic dropout medium lacking Trp, Leu, His and Ade, supplemented with 30 mM 3‐AT. For confirmation of yeast two‐hybrid interactions, subcloning of the full‐length *LRM3* coding sequence, the N‐terminal sequence (amino acids 1–241), the U‐box domain (amino acids 242–305) and the ARM repeat domain (306–841) was performed into the *pGBKT7* vector (Clontech). MYB6 was subcloned into the *pGADT7* vector (Clontech). AD‐MYB6 was then co‐transformed with BD‐LRM3, BD‐LRM3^Nter^, BD‐LRM3^U‐box^ or BD‐LRM3^ARM^ into yeast strain AH109 for further verification of their interactions.

### Pull‐down assay

The full‐length *LRM3* coding sequence in the *pCDFDuet* vector was fused with a MYC tag, while the full‐length *MYB6* coding sequence in *pGEX‐4T‐3* was tagged with a GST tag for pull‐down assay (Gao *et al*., 2017).

### Split‐luciferase complementation assay

The coding sequences of *LRM3* and *MYB6* were amplified and cloned into the *pCAMBIA1300‐35S‐N‐Luc‐RBS* vector and *pCAMBIA1300‐35S‐cLUC‐RBS* vector (Wang *et al*., 2020), respectively. These constructs were transformed into *A. tumefaciens* EHA105 and GV3101 for individual infiltration or co‐infiltration experiments. Infiltration was performed on young, fully expanded *N. benthamiana* leaves using a suspension of mixed *A. tumefaciens* containing each plasmid in an infiltration buffer with OD_600nm_ = 1.0 after incubating for 3 h at room temperature. The surface of the Infiltration leaves was treated with 200 μL of luciferin solution (150 μg/mL). After a 5‐min incubation period, LUC signals were detected under a low‐light cooled CCD camera (Tanon) (Chen *et al*., 2008). For quantification, five leaves were co‐infiltrated for all combinations, and the signal intensity was measured using Image J.

### Co‐immunoprecipitation (Co‐IP) assays

The coding sequence of *LRM3* was amplified and cloned into the *pCAMBIA1300‐35S‐HA‐RBS* vector, and the coding sequence of *MYB6* was amplified and cloned into the *pCAMBIA1300‐35S‐FLAG‐RBS* vector. Both constructs were transformed into *A. tumefaciens* GV3101, and strains constructs were co‐infiltrated into *N. benthamiana* leaf epidermal cells at OD_600 nm_ = 0.6. After 3 d of growth at 25 °C, infiltrated leaves were collected for protein extraction. Total proteins were extracted using an extraction buffer containing 50 mM Tris–HCl (pH 8.0), 120 mM NaCl, 1 mM EDTA, 0.2% (v/v) Triton X‐100, 5 mM DTT, 10% (v/v) glycerol and 1x Roche protease inhibitor cocktail.

The supernatant obtained after centrifugation at 12000 *g* for 15 min at 4 °C was then mixed with anti‐DDDDK‐tag mAb‐Magnetic Beads (MBL) in a volume of 30 μL and incubated at 4 °C for 3 h. Subsequently, the beads were washed five times with washing buffer containing 50 mM Tris–HCl (pH8.0), 120 mM NaCl, 1 mM EDTA, 0.2% v/v Triton X‐100, 5 mM DTT, 10% v/v glycerol and 1× Roche protease inhibitor cocktail. The bound proteins were eluted from the beads using SDS‐PAGE loading buffer and analysed by immunoblotting with anti‐HA or anti‐DDDDK antibodies (MBL). Protein concentration was determined by BCA assay (Solarbio, PC0020). 4%–20% SurePAGE gels (GenScript, M00655) were used to resolve samples for western blot. Primers used to generate the relevant constructs are listed in (Table S2).

### *In vitro* ubiquitination assay

The assay was conducted following previously described procedures with minor modifications. *AtUBC8* and *GmLRM3* were cloned into a modified *pCDFDuet* vector and tagged with S or MYC at the C‐terminus. *AtUBA1* and *GmMYB6* were cloned into a modified *pACYCDuet* vector and tagged with S or 2 × HA at the C‐terminus. *AtUBQ10* was cloned from a plant expression vector into *pET‐28a*, fused to N‐terminal 6 × His and FLAG tags. *Escherichia coli* BL21 (DE3) harbouring different combinations of these expression vectors was cultured in liquid lysogeny broth (LB) at 37 °C. Target protein expression was induced by adding 0.5 mM IPTG when the absorbance at 600 nm reached 0.4–0.6, followed by further growth at 28 °C for 10–12 h and overnight storage at 4 °C. Crude protein extracts were then separated by SDS‐PAGE and subjected to western blotting using corresponding antibodies (Han *et al*., 2017).

### Analysis of MYB6 protein abundance *in vivo*

To assess the abundance of MYB6, GFP alone and MYB6 were cloned into a modified *pCAMBIA1300‐35S‐FLAG‐RBS* vector (Wang *et al*., 2020). The resulting plasmid vector was introduced into *A. rhizogenes* K599 and subsequently transformed into soybean W82 and *lrm3* using a previously described soybean hairy‐root transformation method (Kereszt *et al*., 2007). After 12 days of induction, hairy roots emerged, which were used for assaying protein abundance by western blot.

Total proteins were extracted using an extraction buffer containing 50 mM Tris–HCl (pH 8.0), 120 mM NaCl, 1 mM EDTA, 0.2% (v/v) Triton X‐100, 5 mM DTT, 10% (v/v) glycerol and a Roche Protease inhibitor cocktail at a final concentration of 1×. Following centrifugation at 12 000 *g* for 15 min at 4 °C, the supernatant was mixed with SDS‐PAGE loading buffer (5×) and analysed by western blotting using anti‐DDDDK (MBL) antibody to detect MYB6 fusion proteins and anti‐GFP (MBL) as controls. Signal intensities on blots were quantified using ImageJ software. The protein concentration was determined by BCA protein assay kit (Solarbio, PC0020). 4%–20% SurePAGE gels (GenScript, M00655) were used to resolve samples for western blot. 100 μg of protein was loaded onto gels for protein abundance assay.

### Cell‐free degradation assay

A native protein extraction buffer (50 mM Tris‐MES pH 8.0, 0.5 M sucrose, 1 mM MgCl_2_, 10 mM EDTA pH 8.0 and 5 mM DTT) was used for extracting total proteins from 2‐week‐old W82, *lrm3* and *LRM3 OE* seedlings. Incubations with purified MYB6‐FLAG (1 μg) and crude protein extracts (200 μg), in the presence or absence of 50 μM MG132, supplemented with 1 mM ATP, were performed at 25 °C for varying durations. To terminate the reactions, SDS‐PAGE loading buffer was added to each sample, and protein abundance was detected by western blot using anti‐FLAG antibodies (Cheng *et al*., 2020).

### Protein‐degradation assays

For protein degradation assays, LRM3‐GFP and MYB6‐LUC were transiently expressed in *N. benthamiana* leaves alone or in combination. MG132 (50 mM) dissolved in 0.5% v/v DMSO, or 0.5% v/v DMSO alone as the control, was infiltrated 12 h prior to imaging (Ding *et al*., 2018).

### RNA‐seq and data analysis

For RNA‐seq analysis, 2‐week‐old stems were harvested from W82, *lrm3* and *lrm3* ^*CR1*^. Three RNA‐seq libraries from three biological replicates were generated using a TruSeq RNA Library Prep Kit (Illumina, RS‐122‐9001DOC), followed by sequencing with the Illumina Hiseq X Ten platform at Novogene Biotech Company (Beijing, China) to obtain paired‐end reads with a length of 150 bp. Raw reads were assessed by Fastp (Chen *et al*., 2018). The trimmed reads of each sample mapping to the reference genome employed Hisat2 v.2.0.5 (Kim *et al*., 2019). Gene expression (FPKM, fragments per kilobase of transcript per million fragments mapped) levels were estimated using the Cufflinks software (version v 2.1.1), and differentially expressed genes (DEGs) were selected by using the criteria *q* < 0.05 and |log2 (fold change) | ≥ 1. cDNA was synthesized using a FastQuant RT Kit (with gDNase) (TIANGEN, China), and the instructions of the manufacturer were followed.

### Electrophoretic‐mobility shift assay (EMSA)

EMSA was performed using the Light Shift Chemiluminescent EMSA Kit (Thermo Scientific #20148) following the manufacturer's recommendations. Briefly, recombinant MYB6‐GST protein (1 μg) was incubated with a biotinylated probe in the presence or absence of a cold competitor. Reactions were separated using native gels and transferred to a membrane. Labelled DNA was detected using chemiluminescence. Biotin‐labelled probe and cold competitor probes were synthesized by Sangon Company. For the list of probes, see the supplemental experimental procedures.

### CUT & Tag assay and analysis

Leaf nuclei of MYB6‐Flag overexpressed plants were collected with three replicates per group for the CUT & Tag assay (Vazyme, TD904). Briefly, the cell nucleus was resuspended with wash buffer, incubated with beads, incubated with primary anti‐IgG antibody (1:50, Abcam, ab6892) or anti‐Flag antibody (1:50, MBL, M185‐11R) and incubated with secondary antibodies. Then, the samples were incubated with Hyperactive pA/G‐Transposon Pro, fragmented with MgCl_2_. The DNA was extracted, and PCR amplification was performed using indexing primers (Vazyme, TD202). qPCR was used to detect the enrichment of *PAL1* and *PAL2*. Primers used in CUT & Tag PCR assays are listed in Table S2.

### Haplotype analysis of *LRM3*

For investigation of *LRM3* natural variation, a panel of 1591 sequenced soybean accessions consisting of 277 wild, 405 landrace and 909 cultivated soybeans from NCBI SRA database (https://www.ncbi.nlm.nih.gov) was used for haplotype analysis. Read mapping and variant calling were performed as detailed in (Zhou *et al*., 2021).

A total of 3 SNPs in the 7.8 kb region surrounding *LRM3* were used for haplotype classification. Three main haplotypes were obtained based on qualified genotypic and phenotypic data for *LRM3*. The remaining haplotypes were represented by fewer accessions, were deemed rare and thus were not considered further. Haplotype‐network analysis was performed using PopART based on the ‘Median‐Joining Network’ approach. Phylogenetic analysis was based on NJ tree implemented in MEGA7 software with 5000 bootstraps. Fixation index (*F*_ST_) and nucleotide diversity (*π*) values in wild, landrace and cultivated soybean germplasm were calculated across the ~7.8 kb genomic region of *LRM3* that includes the exons, introns and 5′ and 3′ UTR sequences under a 5 kb sliding window analysis with a 5 kb step using VCFtools (v0.1.14) (Danecek *et al*., 2011).

## Accession numbers

Sequence data from this article can be accessed in *phytozome_13* libraries under accession numbers: *LRM3* (*Glyma.18G055200*), *MYB6* (*Glyma.06G160500*), *PAL1* (*Glyma.10G058200*) and *PAL2* (*Glyma.03G181600*). RNA‐seq data from this article have been submitted to the Genome Sequence Archive (GSA) under accession number CRA024512. The soybean reference genome (*Glycine_max.v2*) was utilized in this study.

## Author contributions

Y.H.Y., Z.Y.C., X.J.Y., K.Q.T., H.Y. and J.S.G. performed experiments. J.T.L., W.Z. and Y.Z. conducted fieldwork. M.R.B., B.F.Z., Z.C.D. and Z.H.Z. analysed data. X.Z.F., S.X.Y., Y.H.Y. and Z.Y.C. wrote the paper. X.Z.F. and S.X.Y. conceived the project.

## Declaration of interests

The authors declare no competing interests.

## Supporting information


**Figure S1** Phenotypic and agronomic traits of *lrm3* mutants.
**Figure S2** Identification of *LRM3* by bulked‐segregant analysis.
**Figure S3** Amino acid alignment between wild‐type W82 and the *lrm3* EMS mutant.
**Figure S4** Phylogeny analysis of LRM3 protein.
**Figure S5** The characterization of differences of *LRM3* gene expression and protein size between W82 and *lrm3* mutant.
**Figure S6** Subcellular localization of LRM3‐GFP.
**Figure S7** Identification of *LRM3* complementation transgenic lines.
**Figure S8** Genotypic characteristics of the *LRM3* CRISPR/Cas9‐edited mutants.
**Figure S9** Genotypic and phenotypic characteristics of the *Glyma.11G161500* CRISPR/Cas9 transgenic line.
**Figure S10** Relative expression of *LRM3* in W82 and *LRM3* overexpression lines.
**Figure S11** CRISPR/Cas9 gene editing confirms *Glyma.18G055200* is *lrm3*, and its function is required for proper soybean growth.
**Figure S12** Expression pattern of MYB6.
**Figure S13** Yeast two‐hybrid assay of the interaction *in vivo* between LRM3 and empty AD vector.
**Figure S14** GFP and LRM3‐GFP abundance in *N. benthamiana* leaves.
**Figure S15** Identification of *MYB6* overexpression transgenic lines.
**Figure S16**. Transcriptomic analysis of W82, *lrm3* mutant, and *lrm3* ^*CR1*^ stems by RNA‐seq.
**Figure S17** Carbohydrate and lignin biosynthesis‐related genes are differentially expressed in the *lrm3* mutant and *lrm3* ^*CR1*^.
**Figure S18** Western blot of GFP and MYB6‐GFP expression in transgenic soybean hairy roots in the wild‐type W82 and *lrm3* mutant backgrounds.
**Figure S19** *PAL1* and *PAL2* are upregulated in *LRM3* overexpression lines.
**Figure S20** MYB6‐GFP suppresses *PAL* promoter activity *in vivo*.
**Figure S21** *LRM3* phylogeny and haplotype‐network analysis.
**Table S1** Chi‐squared test for segregation ration of normal and mutant plants in the F_2_ generation (*lrm3* × Hedou 12).
**Table S2** Primers used for gene expression and vector construction.
**Table S3** The possible interaction proteins of LRM3 by Y2H screening.

## Data Availability

The data that supports the findings of this study are available in the supplementary material of this article.
