# Supplementary material for: LRM3 positively regulates stem lodging resistance by degradating MYB6 transcriptional repressor in soybean
Source: Plant Biotechnol J. 2025 May 7;23(7):2978–93. doi: 10.1111/pbi.70124 (PMC12205893; doi:10.1111/pbi.70124)
Supplement: Supplementary file 1 — Figure S1 Phenotypic and agronomic traits of lrm3 mutants. Figure S2 Identification of LRM3 by bulked‐segregant analysis. Figure S3 Amino acid alignment between wild‐type W82 and the lrm3 EMS mutant. Figure S4 Phylogeny analysis of LRM3 protein. Figure S5 The characterization of differences of LRM3 gene expression and protein size between W82 and lrm3 mutant. Figure S6 Subcellular localization of LRM3‐GFP. Figure S7 Identification of LRM3 complementation transgenic lines. Figure S8 Genotypic characteristics of the LRM3 CRISPR/Cas9‐edited mutants. Figure S9 Genotypic and phenotypic characteristics of the Glyma.11G161500 CRISPR/Cas9 transgenic line. Figure S10 Relative expression of LRM3 in W82 and LRM3 overexpression lines. Figure S11 CRISPR/Cas9 gene editing confirms Glyma.18G055200 is lrm3, and its function is required for proper soybean growth. Figure S12 Expression pattern of MYB6. Figure S13 Yeast two‐hybrid assay of the interaction in vivo between LRM3 and empty AD vector. Figure S14 GFP and LRM3‐GFP abundance in N. benthamiana leaves. Figure S15 Identification of MYB6 overexpression transgenic lines. Figure S16. Transcriptomic analysis of W82, lrm3 mutant, and lrm3 CR1 stems by RNA‐seq. Figure S17 Carbohydrate and lignin biosynthesis‐related genes are differentially expressed in the lrm3 mutant and lrm3 CR1 . Figure S18 Western blot of GFP and MYB6‐GFP expression in transgenic soybean hairy roots in the wild‐type W82 and lrm3 mutant backgrounds. Figure S19 PAL1 and PAL2 are upregulated in LRM3 overexpression lines. Figure S20 MYB6‐GFP suppresses PAL promoter activity in vivo. Figure S21 LRM3 phylogeny and haplotype‐network analysis. Table S1 Chi‐squared test for segregation ration of normal and mutant plants in the F2 generation (lrm3 × Hedou 12). Table S2 Primers used for gene expression and vector construction. Table S3 The possible interaction proteins of LRM3 by Y2H screening. [file PBI-23-2978-s001.docx]

Supplemental data


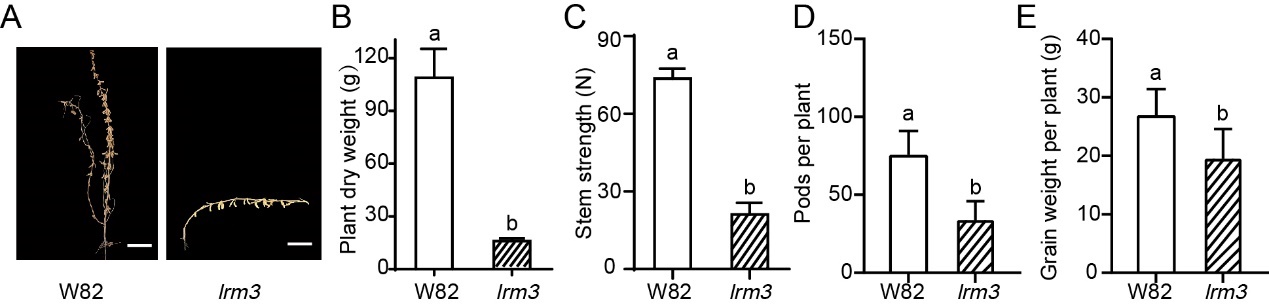


Figure S1. Phenotypic and agronomic traits of *lrm3* mutants.

A. Representative images of field-grown W82 and *lrm3* plants at the R8 stage. Scale bar = 10 cm.

B. Dry weight of W82 and *lrm3* at the R8 stage (full maturity, left), as sampled in D. Data are the mean ± standard deviation of n = 30. Lignin contents at the first internode in W82 and *lrm3* at the V7 stage. Data are the mean ± standard deviation of n = 5. A Student’s t-test (two-sided) was used to generate the *p* values, different lowercase letters indicate statistically significant differences at *p* < 0.05.

C. Rind-penetration strength at the first internode in W82 and *lrm3* at the V7 stage. Data are the mean ± standard deviation of n = 5. A Student’s t-test (two-sided) was used to generate the *p* values, different lowercase letters indicate statistically significant differences at *p* < 0.05.

D, E. Agronomic traits in W82 and *lrm3* at the R8 stage. Pods per plant (D) and Grain weight per plant (E). Data are the mean ± standard deviation of n = 30. A Student’s t-test (two-sided) was used to generate the *p* values, different lowercase letters indicate statistically significant differences at *p* < 0.05. CWR, cell wall residues.


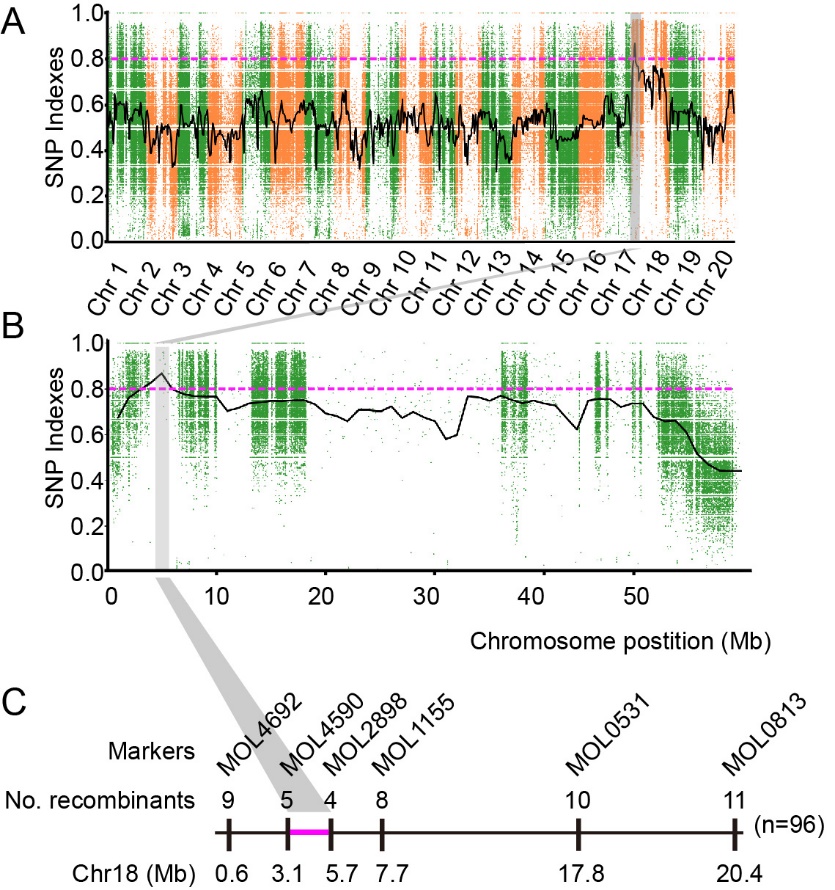


Figure S2. Identification of *LRM3* by bulked-segregant analysis.

A, B. Bulk-segregant analysis mapping of the *LRM3* locus. The SNP indexes across all chromosomes (A) and chromosome 18 (B) of the *lrm3* mutant pool from the F_2_ population are shown. The candidate region for the *lrm3* locus is identified between 3.5-5.5 Mb on chromosome 18 as indicated by the rectangular gray box in B.

C. Summary of the positional cloning of the *LRM3* locus to chromosome 18. The Indel markers MOL4590 and MOL2898 were used for initial mapping.


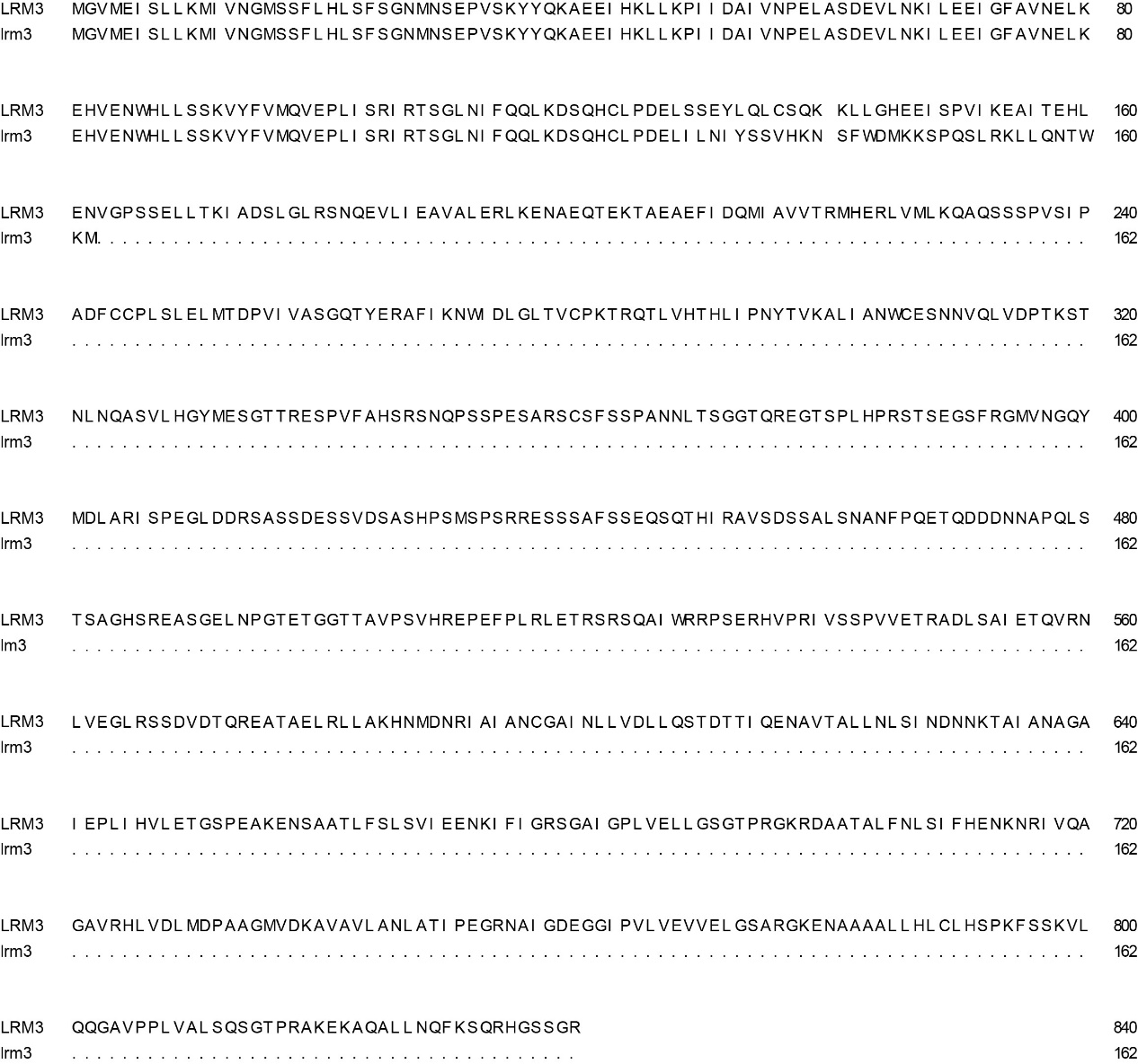


Figure S3. Amino-acid alignment between wild-type W82 and the *lrm3* EMS mutant. The lrm3 protein is truncated at 678 amino acids due to a stop codon resulting from a base substitution in the *lrm3* mutant.


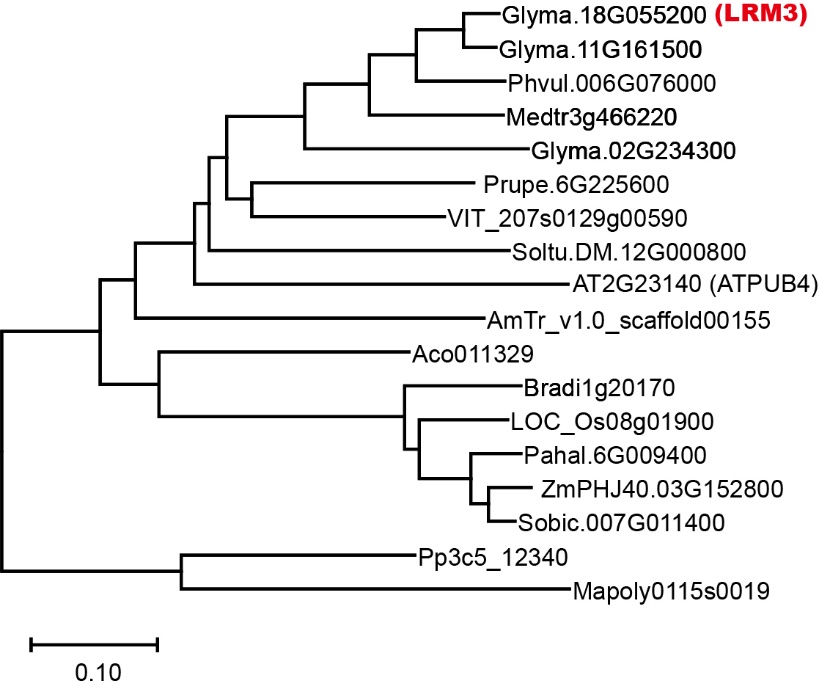


Figure S4. Phylogeny analysis of LRM3 protein.

Neighbor-joining phylogenetic tree of LRM3 homologs. The sequences used for the phylogenetic tree are from *Glycine max*, *Phaseolus vulgaris*, *Medicago truncatula*, *Prunus persica*, *Vitis vinifera*, *Solanum tuberosum*, *Arabidopsis thaliana*, *Zea mays*, *Sorghum bicolor*, *Brachypodium distachyon*, *Panicum hallii*, *Oryza sativa*, *Ananas comosus*, *Marchantia polymorpha*, *Physcomitrium patens* and *Amborella trichopoda.* The phylogenetic tree was constructed using MEGA 7.


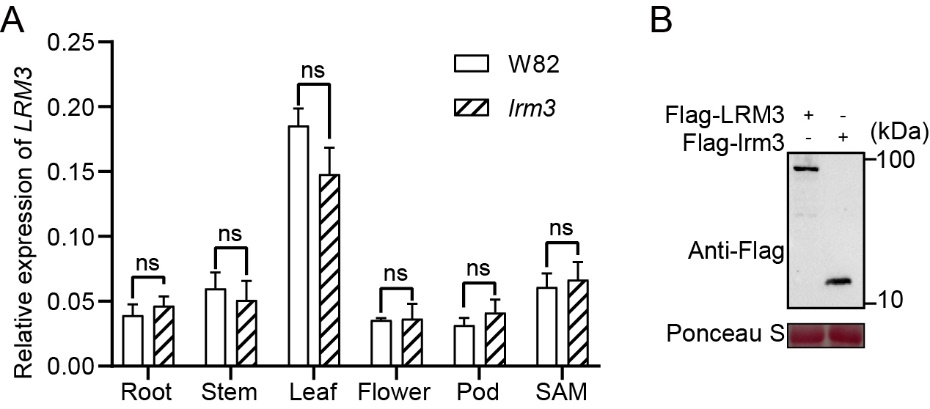


Figure S5. The characterization of differences of *LRM3* gene expression and protein size between W82 and *lrm3* mutant.

A. The expression of *LRM3* gene relative to *CONS4* in different tissues of Williams 82 and *lrm3* mutant. Relative expression data are presented as mean ± standard deviation (n = 3).

B. Western blotting of transiently expressed proteins from Flag-LRM3 and Flag-lrm3 construct in *N. benthamiana* leaves using anti-Flag antibody. In comparison to the wild-type LRM3 protein with a molecular weight of 92.5 kDa, the lrm3 is only 18.5 kDa. Ponceau S staining of Rubisco proteins were used as loading controls.


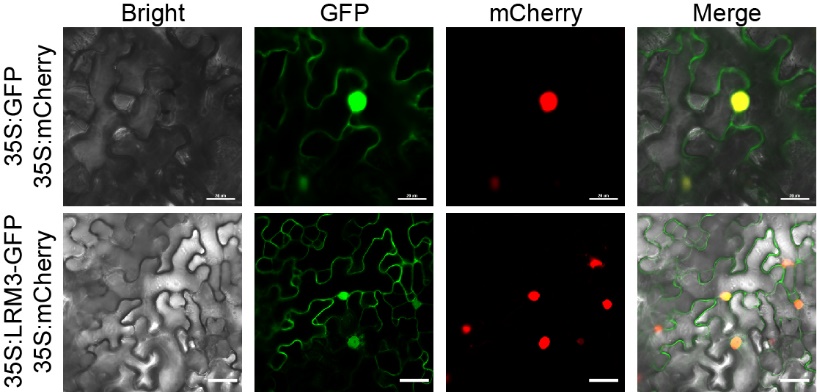


Figure S6. Subcellular localization of LRM3-GFP.

Subcellular localization of LRM3-GFP in *N. benthamiana* cells expressing the *35S::LRM3-GFP* construct. Scale bar, 20 µm.


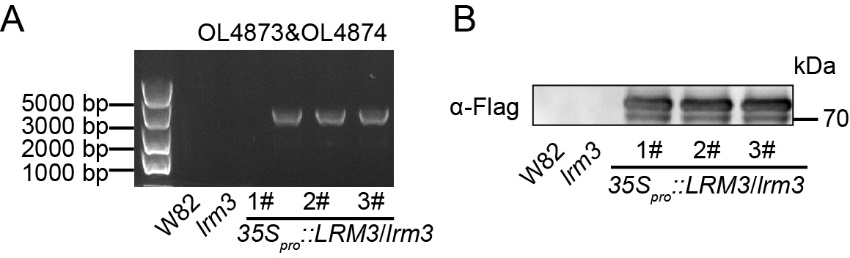


Figure S7. Complementation of *lrm3* in soybean

A. PCR amplification of the vector fragments in the *35Spro::LRM3* construct was conducted to test the complementation lines. W82 and the *lrm3* mutant served as negative controls. The 2829 bp fragments were amplified in complementation transgenic lines using the primers OL4873 and OL4874.

B. Immunoblot analysis was conducted to detect the LRM3-Flag protein using anti-Flag antibodies in the complementation lines. The 92.5 kDa of LRM3-Flag fusion proteins were found in the complementation transgenic lines.


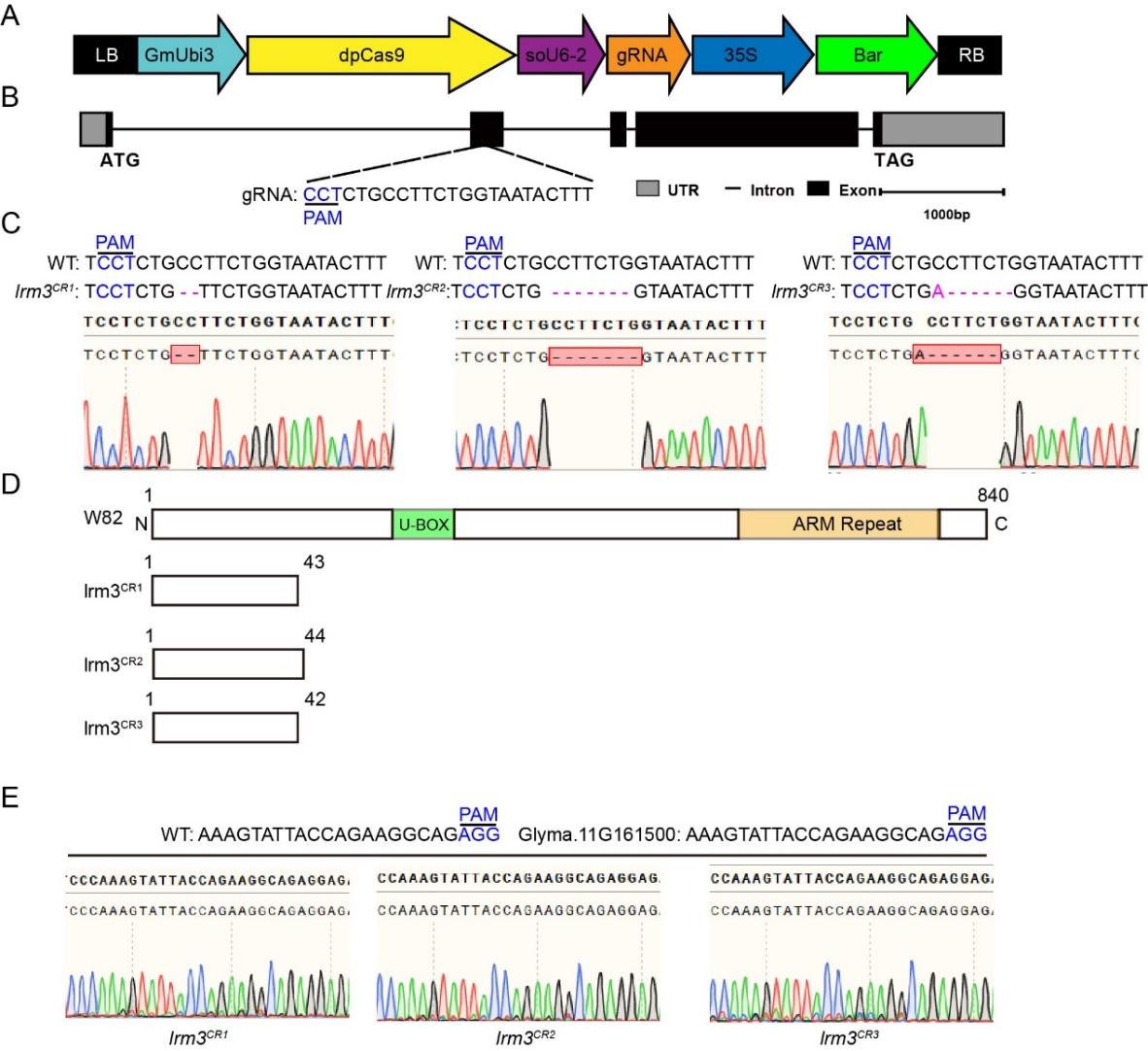


Figure S8. Genotypic characteristics of the *LRM3* CRISPR/Cas9 edited mutants.

A. Structure of the CRISPR/Cas9 vector T-DNA region.

B. Genomic structure of *LRM3* and the sgRNA site targeting it.

C. Genotype of CRISPR-induced *lrm3* mutants in the T_2_ generation. Nucleotides in blue represent the PAM motif of the target site. Nucleotides substituted are shown in pink.

D. Domain representations of the wild-type Glyma.18G055200 and lrm3^CR^ proteins.

E. Detection of *LRM3* homologs off-target mutations.


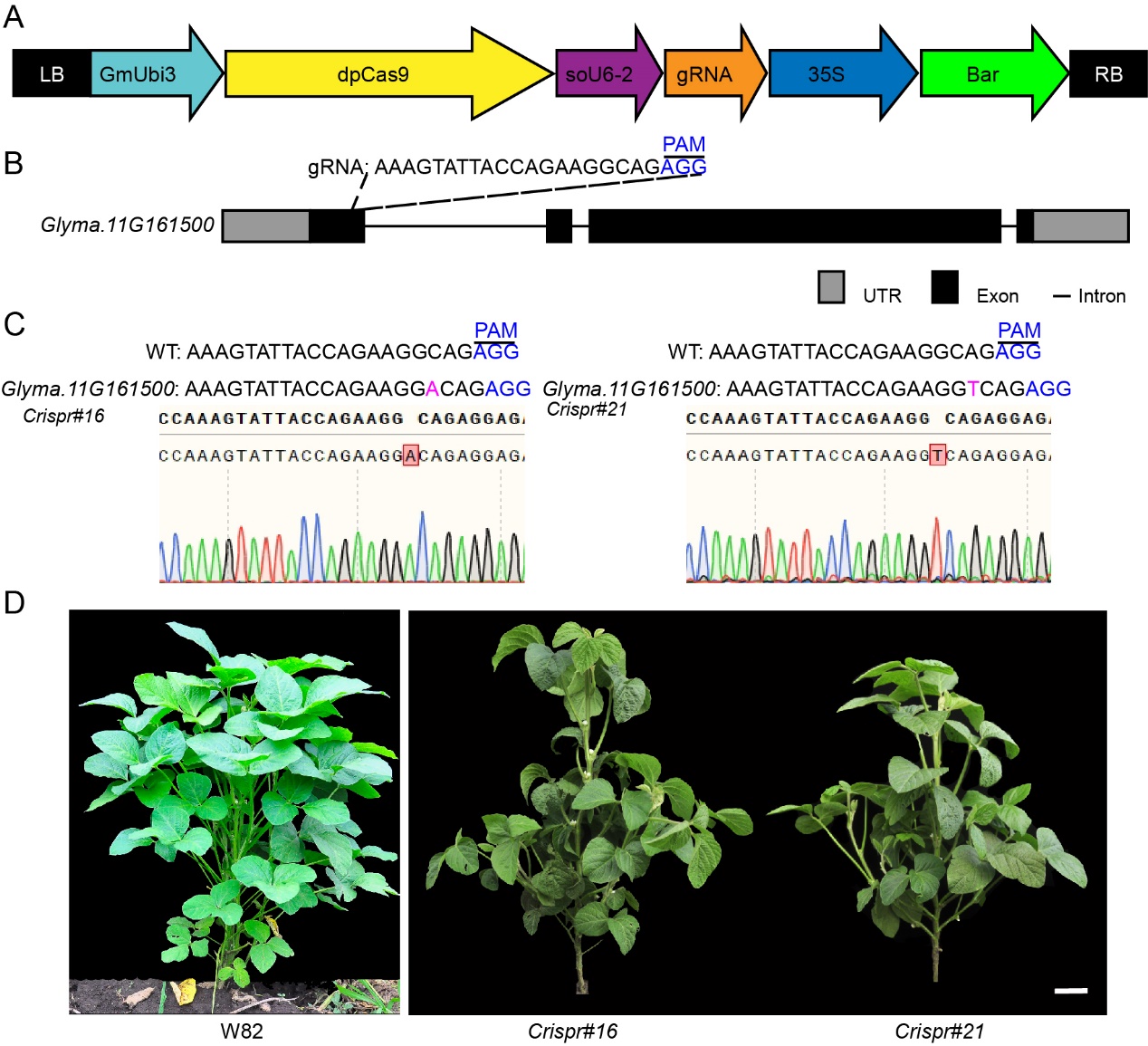


Figure S9. Genotypic and phenotypic characteristics of the *Glyma.11G161500* CRISPR/Cas9 transgenic line.

A. Summary of the CRISPR/Cas9 vector T-DNA region used for gene editing.

B. Genomic structure of *Glyma.11G161500* and the sgRNA site used to edit it.

C. Genotypes of CRISPR *Glyma.11G161500* mutants in the T_2_ generation. Nucleotides in blue represent the PAM motif of the target site. Nucleotides substituted are shown in pink.

D. Representative images of wild-type W82 and *Glyma.11G161500* knockout mutants generated through CRISPR/Cas9 gene editing. Scale bar = 5 cm.


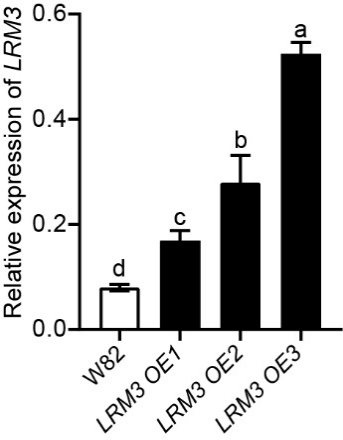


Figure S10. Relative expression of *LRM3* in W82 and *LRM3* overexpression lines.

qRT-PCR of *LRM3* expression in the wild-type W82 and *LRM3* overexpression lines. Data are shown as mean ± standard error of three biological replicates in one experiment and relative expression was normalized to *CONS4*. A one-way ANOVA was used to generate the *p* values, different lowercase letters indicate statistically significant differences at *p* < 0.05.


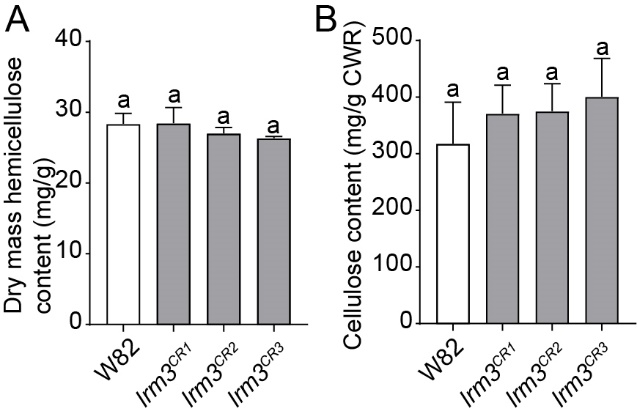


Figure S11. CRISPR/Cas9 gene editing confirms *Glyma.18G055200* is *lrm3* and its function is required for proper soybean growth.

A, B. Hemicellulose (A) and cellulose (B) contents at the first internode in W82 and *lrm3^CR^s* at the V7 stage. Data are means ± standard deviation from n = 5. A one-way ANOVA was used to generate the *p* values, different lowercase letters indicate statistically significant differences at *p* < 0.05. CWR, cell wall residues.


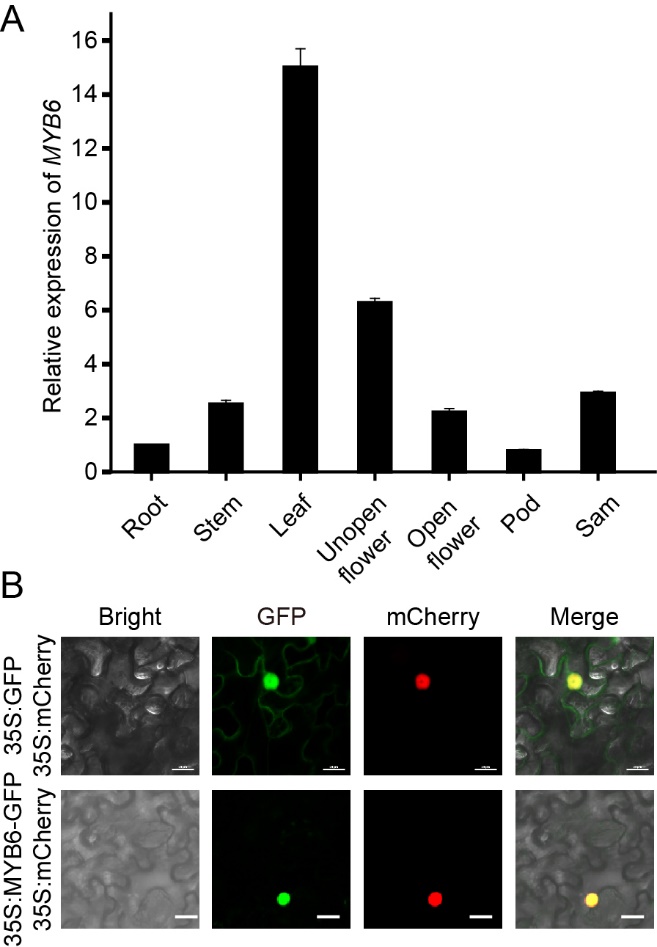


Figure S12. Expression pattern of MYB6*.*

A. Relative *MYB6* expression levels in different tissues in Williams 82. Relative expression data are presented as mean ± standard deviation (n = 3).

B. Subcellular localization of MYB6-GFP in *N. benthamiana* cells expressing a 35Spro:MYB6–GFP construct. Scale bar = 20 µm.


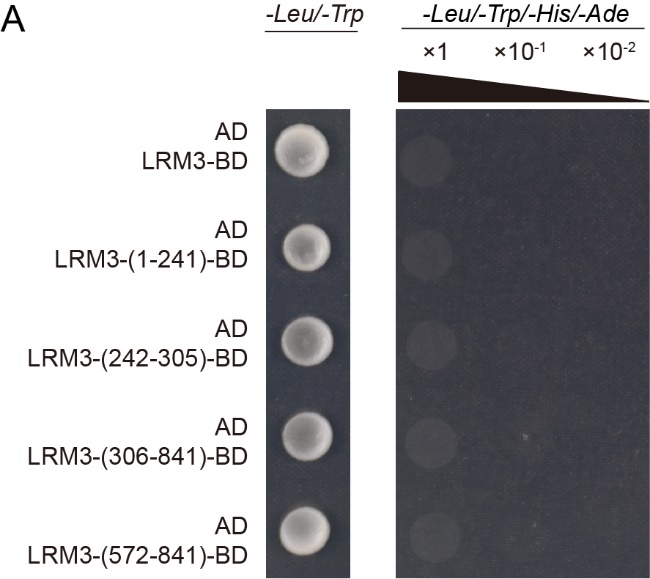


Figure S13. Yeast two-hybrid assay of the interaction *in vivo* between LRM3 and empty AD vector.

A. Transformed cells were grown on a synthetic medium lacking Trp and Leu (-Trp/-Leu) or Trp, Leu, His, and Ade (-Trp/-Leu/-His/-Ade). The numbers above the colony images indicate two serial dilutions. BD, GAL4 DNA-binding domain; AD, GAL4 activation domain.


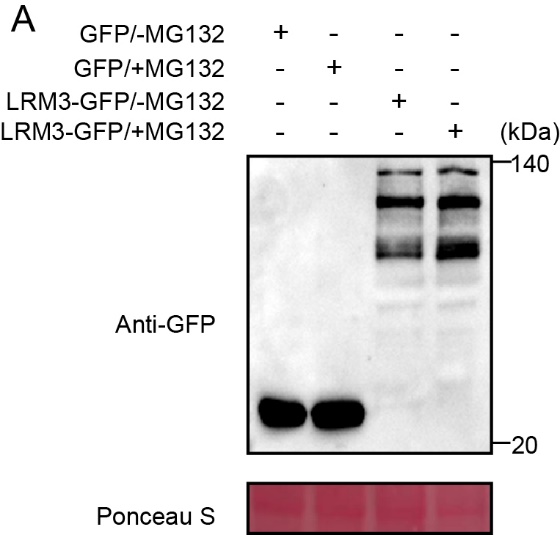


Figure S14. GFP and LRM3-GFP abundance in *N. benthamiana* leaves.

A. Total proteins were extracted from *N. benthamiana* leaves as shown in Fig. 3J and Ponceau S was used as a loading control.


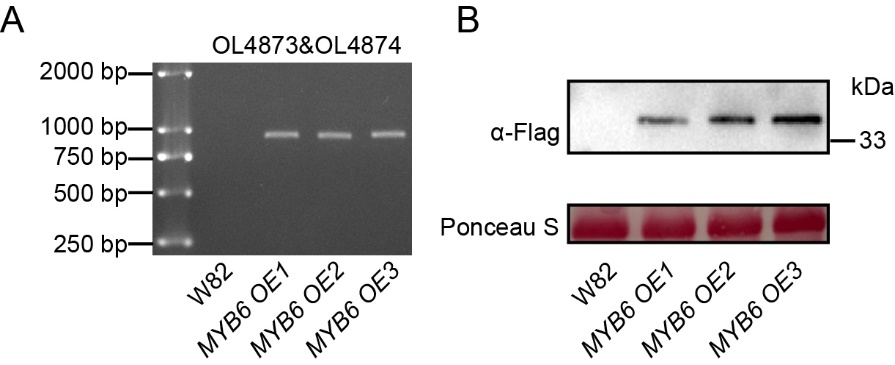


Figure S15. Identification of *MYB6* overexpression transgenic lines.

A. PCR amplification of the vector fragments in the *35S_pro_::MYB6* construct was performed to test the overexpression lines, with W82 serving as a negative control.

B. Immunoblot analysis was conducted to detect the MYB6-Flag protein using anti-Flag antibodies in the overexpression lines. The 38.5 kDa of MYB6-Flag fusion proteins were found in the overexpression transgenic lines.


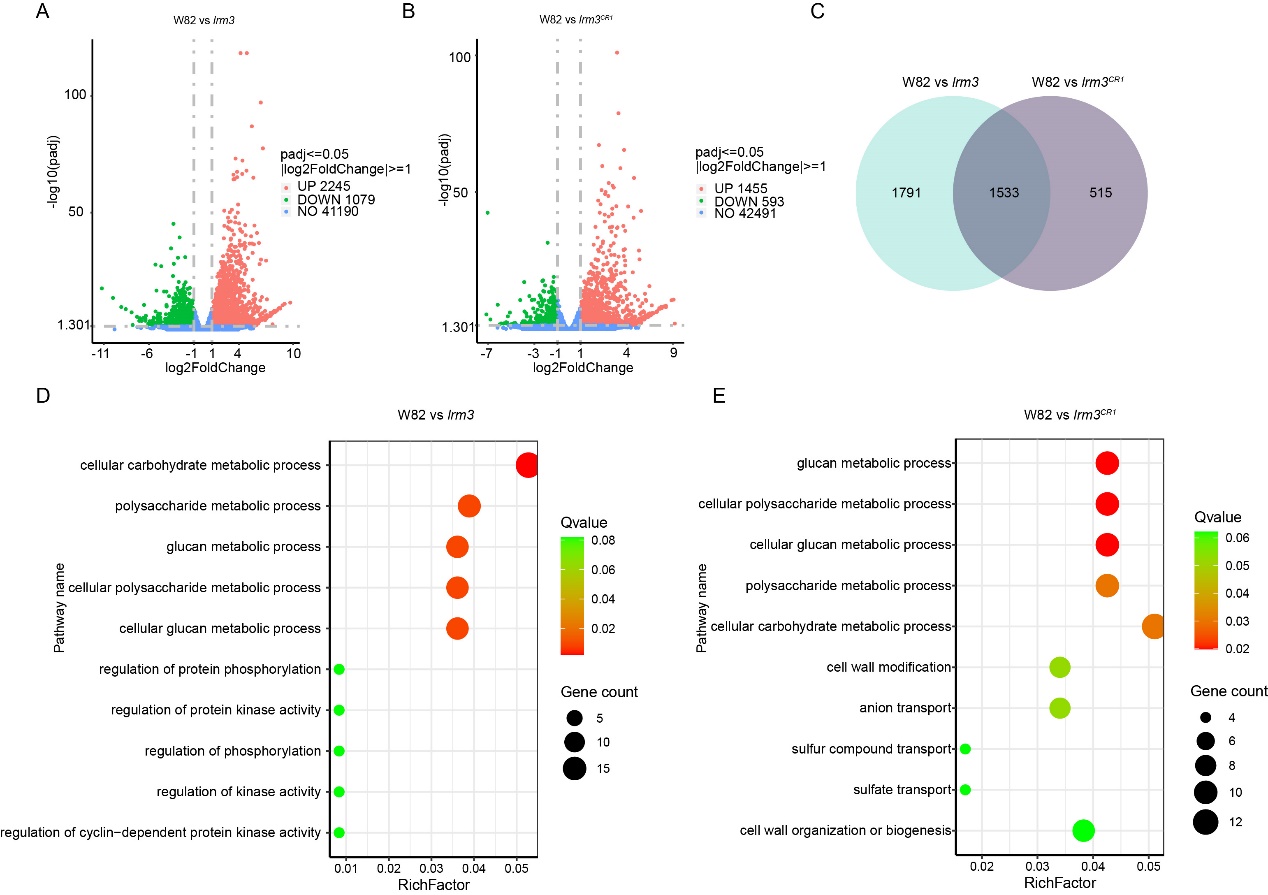


Figure S16. Transcriptomic analysis of W82, *lrm3* mutant and *lrm3^CR1^* stems by RNA-seq.

A. Volcano plots of up-regulated and down-regulated genes between W82 and *lrm3* mutant. Statistically significantly up-and down-regulated genes were defined as those with fold change > 2 and adjusted *p* < 0.05.

B. Volcano plots of up-regulated and down-regulated genes between W82 and *lrm3^CR1^* mutant. Statistically significantly up-and down-regulated genes were defined as those with fold change > 2 and adjusted *p* < 0.05.

C. Venn diagram of overlapping genes between W82 vs. *lrm3* mutant and W82 vs. *lrm3^CR1^*.

D, E. GO pathway-enrichment analysis of down-regulated genes from (A) and (B). The sphere’s size is proportional to gene count, whereas the hue of the sphere is indicative of the magnitude of the *p* value.


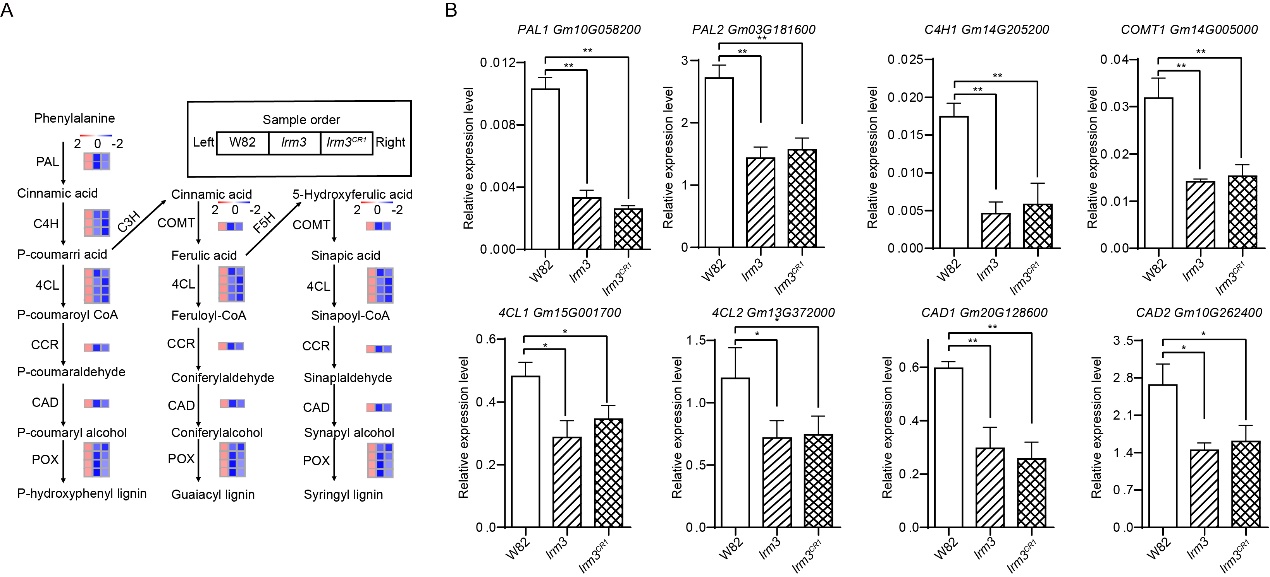


Figure S17. Carbohydrate and lignin biosynthesis related genes are differentially expressed in the *lrm3* mutant and *lrm3^CR1^*.

A. Transcript profiling of DEGs in the phenylpropanoid biosynthesis pathway in W82, *lrm3* mutant, and *lrm3^CR1^.* PAL: phenylalanine ammonia-lyase; C4H: cinnamate 4-hydroxylase; 4CL: 4-coumarate: CoA ligase; C3H: p-coumarate 3 hydroxylase; CCR: cinnamoyl CoA reductase; CAD: cinnamyl alcohol dehydrogenase; COMT: caffeic acid 3-O-methyltransferase, POX: peroxidase.

B. qRT-PCR validation of select phenylpropanoid biosynthesis genes. Data are shown as mean ± standard error of three biological replicates in one experiment and relative expression was normalized to *CONS4*. A one-way ANOVA was used to generate the *p* values, different lowercase letters indicate statistically significant differences at *p* < 0.05.


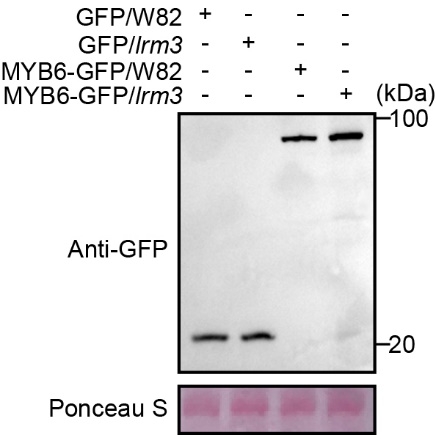


Figure S18. Western blot of GFP and MYB6-GFP expression in transgenic soybean hairy roots in the wild-type W82 and *lrm3* mutant backgrounds. Ponceau S staining of the membrane was used as a loading control.


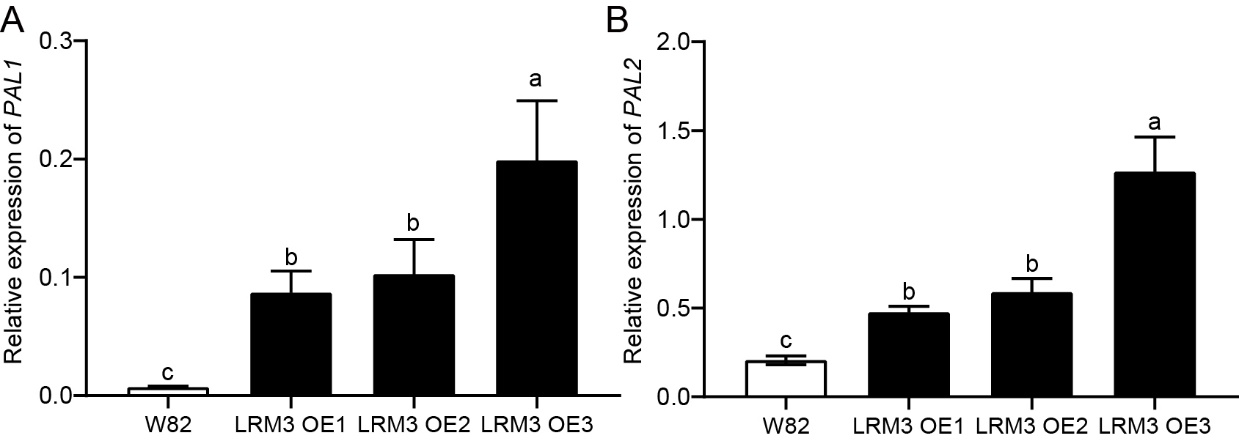


Figure S19. *PAL1* and *PAL2* are up-regulated in *LRM3* over-expression lines.

A, B. qRT-PCR of *PAL1* (A) and *PAL2* (B) expression in the wild-type W82 and stable-transgenic *LRM3* over-expression lines. Data are shown as mean ± standard error of three biological replicates in one experiment and relative expression was normalized to *CONS4*. A one-way ANOVA was used to generate the *p* values, different lowercase letters indicate statistically significant differences at *p* < 0.05.


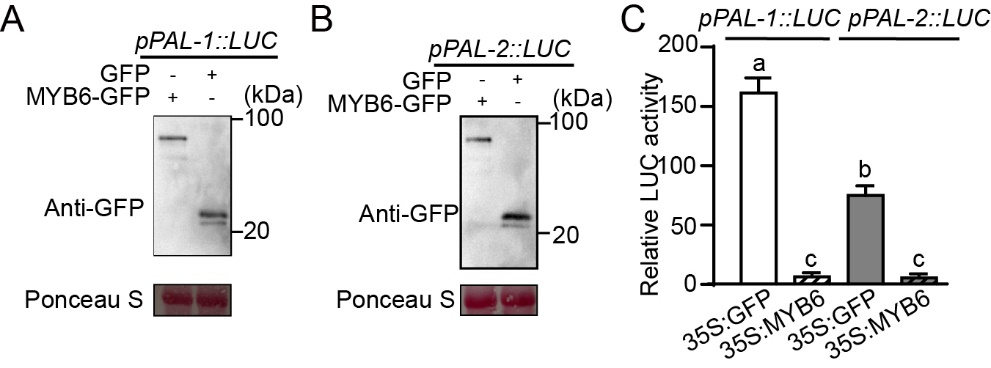


Figure S20. MYB6-GFP suppresses *PAL* promoter activity *in vivo*.

A, B. Abundance of GFP and MYB6-GFP in *N. benthamiana* leaves. Ponceau S as a loading control.

C. Relative LUC activity as captured in Fig. 4G, H was quantified and shown as means ± standard error of three technical replicates of one experiment. Different lowercase letters indicate statistically significant differences at p < 0.01 by one-way ANOVA.


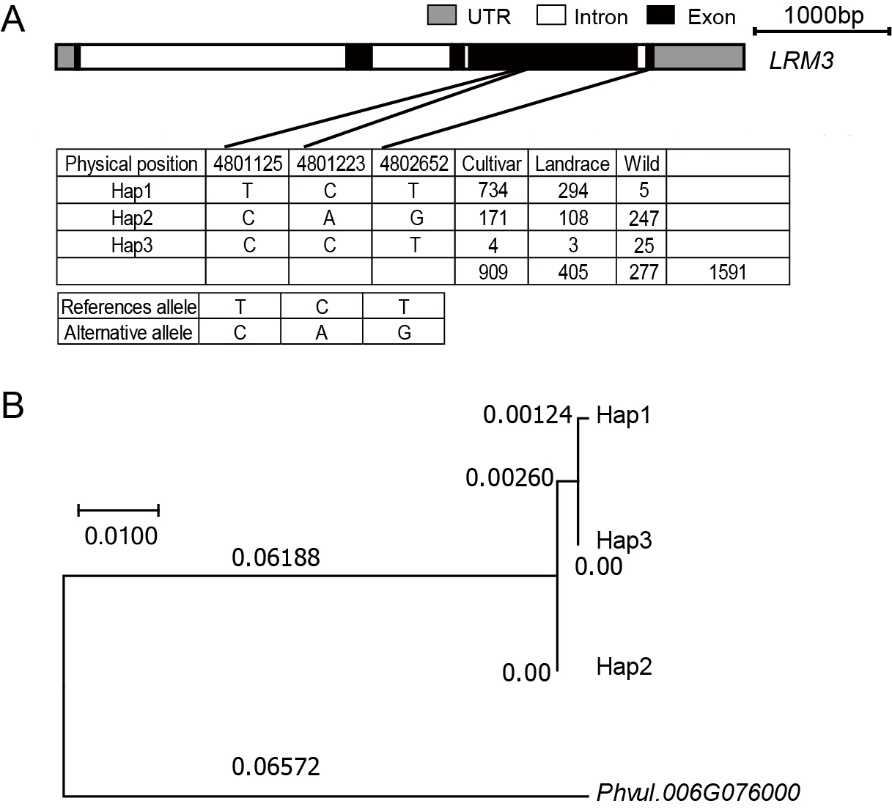


Figure S21. *LRM3* phylogeny and haplotype-network analysis.

A. *LRM3* haplotypes in natural populations. Top: Schematic of the *LRM3* genomic structure; gray, untranslated regions (UTRs); black, exons; white, introns. Bottom: *LRM3* polymorphisms across accessions relative to the W82 reference genome (Hap3). The number of varieties bearing each haplotype (Hap1-3) is shown to the right.

B. Evolutionary relationships between the *LRM3* haplotypes, Hap1-3. The optimal tree with the sum of branch length = 0.13144202 is shown. The tree is drawn to scale, with branch lengths in the same units as those of the evolutionary distances used to infer the phylogenetic tree. The evolutionary distances were computed using the Poisson correction method. Evolutionary analyses were conducted in MEGA7.

Table S1. Chi-squared test for segregation ration of normal and mutant plants in the F_2_ generation (*lrm3* × Hedou 12)

| Cross | Generation | No. of plants |  |  |  |  |  |  |
| --- | --- | --- | --- | --- | --- | --- | --- | --- |
|  |  | Total | Wild type | Mutant | Expected ratio | χ^2^ | *p* | *df* |
| *lrm3 ×* Hedou 12 | F_2_ | 189 | 145 | 44 | 3:1 | 0.153 | 1 | 0.696 |

Table S2. Primers used for gene expression and vector construction.

| Primer Name | Forward |
| --- | --- |
| MOL4692_ mapping_F | CACCTTTTGTTTGGTTA |
| MOL4692_ mapping_R | TTATTAGCTTTTTTGTCCGCC |
| MOL4590_mapping_F | CAGCAGGAGAAATCTGTGGAGT |
| MOL4590_mapping_R | ATGAACAACTCAATGCACCACC |
| MOL2898_mapping_F | GTTTTAAGACTAATGTCAGGGTTCG |
| MOL2898_mapping_R | GTATAGTGCGTCAACTCAGAAGTAAGA |
| MOL1155_mapping_F | GTTGGTTCTTGTGGTAGGGGTTA |
| MOL1155_mapping_R | AGCGCTTGGAGAACTTTACTCTATT |
| MOL0531_mapping_F | TGGCTTGTCTGTCTTTGG |
| MOL0531_mapping_R | ATCCCATCATGTTCTCCTCT |
| MOL0813_mapping_F | TCCTTCTTGCCTTGTTTCTATCAC |
| MOL0813_mapping_R | TCTCTAATAGGGTGGGAATCACTT |
| MOL4704_mapping_F | ATATTTCGTAGCCTGCAG |
| MOL4704_mapping_R | CACGACTATTGTTTAGAGGAA |
| MOL4583_mapping_F | TCCCATTGTTTCCTTCGTCCTA |
| MOL4583_mapping_R | GTGTGAATGCACGTATCAATCACAA |
| MOL4589_mapping_F | ACCACCCCTTATTCTTGGACCT |
| MOL4589_mapping_R | GCAACATTCCACGTTCTCAAAT |
| MOL4001_mapping_F | TACATTAAATATACATTAAATTTTTTATTG |
| MOL4001_mapping_R | AAAATTATCTCTGACAAATTTA |
| MOL4002_mapping_F | TGCACCAAAATTAATTGTCACAAAATTT |
| MOL4002_mapping_R | ATCGATGTGTTTTTTAGTAATTGG |
| MOL4003_mapping_F | AATTTCCTACAATATTCAGCACCTAATATT |
| MOL4003_mapping_R | CCCTATTTGAGTTTTGTTTTGATTGCC |
| MOL4591_mapping_F | GTTTGCTTCGGTTGCGATGT |
| MOL4591_mapping_R | AACGAGTAAACGCATCCCTGAT |
| *Glyma.*10G058200_qPCR_F | ACCACAACTGCGACCGATAG |
| *Glyma.*10G058200_qPCR_R | TCCATGTTAACAAAGAGGTCACA |
| *GmLRM3*_qPCR_F | TCAGGCTCTCCTCAATCAATTT |
| *GmLRM3*_qPCR_R | CTTTCCAAGCAAGAGAAAACGA |
| Actin_qPCR_F | GATCAGCAATTATGCACAACG |
| Actin_qPCR_R | CCGCCACCATTCAGATTATGT |
| Actin_qPCR_F | GCAAGTGGTCGTACAACTGG |
| Actin_qPCR_R | ACCAGCCAGATCAAGACGAA |
| *Glyma.03G181600*_qPCR_F | GATGTGAACTCGTCCATCTTTC |
| *Glyma.03G181600*_qPCR_R | CAACCCAGTCCCTAATTCCTC |
| *Glyma.10G058200*_qPCR_F | ACAAGGGCACTGACAGCTAC |
| *Glyma.10G058200*_qPCR_R | TGTGGGGTAGGGTGCAATTG |
| *Glyma.14G205200*_qPCR_F | CATGACCGTCCCCTTCTTCA |
| *Glyma.14G205200*_qPCR_R | TCGGGGTTGTTCTTGACGTC |
| *Glyma.14G005000*_qPCR_F | TGGCTTTGAGGGTGTTGGAT |
| *Glyma.14G005000*_qPCR_R | GCTTCTTTGATGACGTGGGG |
| *Glyma.15G001700*_qPCR_F | CCGACACAACCCAGGATGAA |
| *Glyma.15G001700*_qPCR_R | ACGGTGACTTGGGAATTGCA |
| *Glyma.13G372000*_qPCR_F | TCCAGCTGAACTTGAAGCCC |
| *Glyma.13G372000*_qPCR_R | CATCCTCGGTTGTGTCGGTAT |
| *Glyma.20G128600*_qPCR_F | CAGAGCAAGTGGCACCATTAC |
| *Glyma.20G128600*_qPCR_R | GATTGACCTCCTCCCTAGCAT |
| Glyma.18G055200_Crispr_F | ATTAAAGTATTACCAGAAGGCAG |
| Glyma.18G055200_Crispr_R | AACCTGCCTTCTGGTAATACTTT |
| *Glyma.11G161500_F* | ATTAAAGTATTACCAGAAGGCAG |
| *Glyma.11G161500_R* | AACCTGCCTTCTGGTAATACTTT |
| *GmLRM3*_pGBKT7*_F* | CATGGAGGCCGAATTCATGGGTGTTATGGAGATATCATTGT |
| *GmLRM3*_pGBKT7*_R* | GCAGGTCGACGGATCCTCAGCCCCTCCCAGAACTCC |
| *GmLRM3*_pGBKT7(1~241)*_F* | CATGGAGGCCGAATTCATGGGTGTTATGGAGATATCATTGT |
| *GmLRM3*_pGBKT7(1~241)*_R* | ctgcaggtcgacggatccAGCAGGTATTGAAACTGG |
| *GmLRM3*_pGBKT7(242~305)*_F* | ccatggaggccgaattcatgGATTTTTGTTGTCCTC |
| *GmLRM3*_pGBKT7(242~305)*_R* | gcaggtcgacggatccCCAATTTGCAATTAGTGC |
| *GmLRM3*_pGBKT7(306~840)*_F* | ccatggaggccgaattcatgTGTGAATCAAACAATG |
| *GmLRM3*_pGBKT7(306~840)*_R* | GCAGGTCGACGGATCCTCAGCCCCTCCCAGAACTCC |
| *GmLRM3*_pGBKT7(525~840)*_F* | ccatggaggccgaattcatgCAAGCCATTTGGAGGC |
| *GmLRM3*_pGBKT7(525~840)*_R* | GCAGGTCGACGGATCCTCAGCCCCTCCCAGAACTCC |
| *Glyma.20G161000*_pGADT7*_F* | GCCATGGAGGCCAGTGAATTCATGCCAGTTGCAAAACTCC |
| *Glyma.20G161000*_pGADT7*_R* | CAGCTCGAGCTCGATGGATCCCTAGGTTGTTGCTTTTTGTTTG |
| *Glyma.17G101100*_pGADT7*_F* | GCCATGGAGGCCAGTGAATTCGGAAGCAGAAGTGGATGAACC |
| *Glyma.17G101100*_pGADT7*_R* | CAGCTCGAGCTCGATGGATCCTCATTGGATTCTACACCCCC |
| *Glyma.08G120600*_pGADT7*_F* | GCCATGGAGGCCAGTGAATTCATGGCTTTTCCAAACCAATC |
| *Glyma.08G120600*_pGADT7*_R* | CAGCTCGAGCTCGATGGATCCTCAAACAAATTGAAGGGACATG |
| *Glyma.07G046000*_pGADT7*_F* | GCCATGGAGGCCAGTGAATTCATGACCAACCCAAAACTAGGCT |
| *Glyma.07G046000*_pGADT7*_R* | CAGCTCGAGCTCGATGGATCCTCACAAGGTGGAATCAAACTCC |
| *Glyma.15G251600*_pGADT7*_F* | GCCATGGAGGCCAGTGAATTCATGGCAGATGAGGTGGTTCTG |
| *Glyma.15G251600*_pGADT7*_R* | CAGCTCGAGCTCGATGGATCCGCTCCAACCTACTCAATGCCT |
| *Glyma.06G160500*_pGADT7*_F* | atggaggccagtgaattcATGGGAAGATCCCCTTGT |
| *Glyma.06G160500*_pGADT7*_R* | cagctcgagctcgatggatccAATGTTCATGGGTCTGTAAA |
| *Glyma.18G125500*_pGADT7*_F* | GCCATGGAGGCCAGTGAATTCATGGTGAAAGGTCCAGGCCT |
| *Glyma.18G125500*_pGADT7*_R* | CAGCTCGAGCTCGATGGATCCCGAGAAGCATCCCATATATCACT |
| *GmLRM3*_pGWB5-GFP*_F* | GCTTTGACTTTGGGGTACCATGGGTGTTATGGAGATATCATTG |
| *GmLRM3*_pGWB5-GFP*_R* | CTAGAGACTTTGCCTCGAGGCCCCTCCCAGAACTCC |
| *GmMYB6*_pGWB5-GFP*_F* | GCTTTGACTTTGGGGTACCATGGGAAGATCCCCTTGTTGCG |
| *GmMYB6*_pGWB5-GFP*_R* | CTAGAGACTTTGCCTCGAGAATGTTCATGGGTCTGTA |
| *GmMYB6*_C-LUC*_F* | ACGGGGGACGAGCTCGGTACCATGGGAAGATCCCCTTGTTGCG |
| *GmMYB6*_C-LUC*_R* | CGCGTACGAGATCTGGTCGACAATGTTCATGGGTCTGTA |
| *GmLRM3*_N-LUC*_F* | ACGGGGGACGAGCTCGGTACCATGGGTGTTATGGAGATATCATTG |
| *GmLRM3*_N-LUC*_R* | AACATCGTATGGGTAGTCGACGCCCCTCCCAGAACTCC |
| *GmLRM3*_pCAMBIA1300-HA*_F* | ACGGGGGACGAGCTCGGTACCATGGGTGTTATGGAGATATCATTG |
| *GmLRM3*_pCAMBIA1300-HA*_R* | AACATCGTATGGGTAGTCGACGCCCCTCCCAGAACTCC |
| *GmMYB6*_pCAMBIA1300-Flag*_F* | ACGGGGGACGAGCTCGGTACCATGGGTGTTATGGAGATATCATTG |
| *GmMYB6*_pCAMBIA1300-Flag*_R* | ATGGTCTTTGTAGTCGTCGACGCCCCTCCCAGAACTCC |
| *GmLRM3*_pACYCDuet-Myc*_F* | tcaccaagccagggatccATGGGTGTTATGGAGATA |
| *GmLRM3*_pACYCDuet-Myc*_R* | ctgctcaggggcgcgccGCCCCTCCCAGAACTCCCA |
| *GmMYB6*_pGEX4T-GST*_F* | GCGTGGATCCCCGAATTCATGGGAAGATCCCCTTGT |
| *GmMYB6*_pGEX4T-GST*_R* | GATGCGGCCGCTCGAGAATGTTCATGGGTCTGTAAA |
| *GmMYB6*_pCAMBIA1300-LUC*_F* | atgaccatgattacgaattcATGGGAAGATCCCCTTGTTG |
| *GmMYB6*_pCAMBIA1300-LUC*_R* | gtcttccatggtaccgagctcAATGTTCATGGGTCTGTAAA |
| *GmMYB6*_pCDFDuet-HA*_F* | atttcagaattcggatccATGGGAAGATCCCCTTGT |
| *GmMYB6*_pCDFDuet-HA*_R* | cgtatgggtaaggcctAATGTTCATGGGTCTGTAAA |
| *pGlyma.10G058200*_pCAMBIA1300-LUC*_F* | atgaccatgattacgaattcCTATGATTATATCACAATCT |
| *pGlyma.10G058200*_pCAMBIA1300-LUC*_R* | cttccatggtaccgagctcCTTAATACAAGAGAATGCAAG |
| pGlyma.03G181600_pCAMBIA1300-LUC_F | gaccatgattacgaattcTCTACAAACAGATTTTTATATT |
| pGlyma.03G181600_pCAMBIA1300-LUC_R | pGlyma.03G181600_pCAMBIA1300-LUC_R |
| *GmPAL1*-CUT&Tag_F | ATCCTGCCCCGTAATCGAACG |
| *GmPAL1*-CUT&Tag_R | ATGAAGAGTGCTGGGGGCAG |
| *GmPAL2*-CUT&Tag_F | GAGAAAGTACCATTCCGACGG |
| *GmPAL2*-CUT&Tag_R | TTGTGGATTTCTTGAGGGGA |

Table S3. The possible interaction proteins of LRM3 by Y2H screening.

| Gene ID | Description |
| --- | --- |
| Glyma.20G161000 | C2H2-LIKE ZINC FINGER PROTEIN |
| Glyma.17G101100 | HNH endonuclease (HNH) |
| Glyma.08G120600 | FLORAL HOMEOTIC PROTEIN AGAMOUS |
| Glyma.07G046000 | PECTINESTERASE 2-RELATED |
| Glyma.06G160500 | MYB TRANSCRIPTION FACTOR-RELATED |
| Glyma.15G251600 | GLUTATHIONE S-TRANSFERASE U21-RELATED |
| Glyma.18G125500 | VOLTAGE-DEPENDENT ANION-SELECTIVE CHANNEL |
